# Ion Channels and Morphology Drive Widespread Resonance in Human Cortical Interneurons

**DOI:** 10.21203/rs.3.rs-11136357/v1

**Published:** 2026-10-12

**Authors:** Huibert Mansvelder, Natia Shamugia, Robin Weiler, Tim Heistek, Kamiliam Nasirova, Sarah Scott, Sara Vargas, Brian Lee, Rachel Dalley, Brian Kalmbach, Jonathan Ting, Ed Lein, Natalia Goriounova

## Abstract

Cortical neurons respond preferentially to specific network oscillation frequencies—a phenomenon driven by membrane potential resonance. While well studied in human pyramidal neurons, adult human interneuron resonance remains largely unexplored. Using Patch-seq, acute electrophysiology, organotypic cultures, and computational modeling, we investigated the mechanisms underlying resonance across human LAMP5, VIP, PV, and SST interneurons. Resonance is significantly more prevalent in human versus mouse interneurons, peaking in parvalbumin (PV) cells. Unlike human pyramidal neurons, interneuron resonance is independent of cortical depth. Instead, resonance frequency and strength strongly correlate with low input resistance, prominent membrane voltage sag, and enriched expression of HCN1, KCNJ16, and CACNA1A ion channel genes. HCN blockade abolished resonance, while enhanced HCN-mediated sag increased resonance prevalence and strength. Finally, computational models revealed that human morphological features and ion channels sharpen frequency tuning, translating into distinct suprathreshold output under noisy, in vivo-like conditions. These findings reveal fundamental species differences governing human cortical microcircuit dynamics.

### Introduction

Uncovering the cellular and circuit mechanisms underlying human cortical function is a central goal of neuroscience. Human cortex features neuron types with specialized biophysical properties that enhance neuronal computational efficiency, and the human inhibitory microcircuit has undergone profound evolutionary adaptations[1–4]. The relative proportion of cortical interneurons is 2.5-fold higher in humans than in mice[5,6], accompanied by increased interneuron connectivity[7], strengthened synaptic drive[8–11], and human-specific di-synaptic inhibition motifs[9]. These specializations may help large human brains operate efficiently on temporal scales comparable to smaller rodent brains[12]. However, how individual human interneurons contribute to cortical network dynamics through intrinsic membrane properties is poorly understood.

In addition to network-level connectivity, cortical network oscillations are shaped by subthreshold membrane potential resonance[13–17], a frequency-tuning mechanism where neurons selectively amplify inputs within a preferred frequency band[18]. Theta-band resonance is prominent in human and non-human primate pyramidal neurons, representing a major divergence from rodents[19–21]. Yet, while species differences in excitatory pyramidal neurons are well documented[19,21], subthreshold resonance in human cortical interneurons remains largely unexplored, leaving a critical gap in our understanding of microcircuit function.

Subthreshold resonance arises from the interplay between passive membrane properties (low-pass filtering) and slow, voltage-dependent active conductances (high-pass filtering)[18,22–24]. A prominent active conductance driving resonance is the hyperpolarization-activated cyclic nucleotide-gated (HCN) channel current ($I_h$)[22]. During hyperpolarization, HCN channels open and inward $I_h$ pushes membrane voltage to more positive potential, producing a characteristic voltage sag and acting as a biological high-pass filter. Human interneurons, particularly fast-spiking parvalbumin (PV) cells, exhibit significantly elevated somatic HCN expression with strong $I_h$ compared to mouse counterparts[2,4]. This raises the hypothesis that human interneurons display robust, specialized resonant properties, but this has not been tested. Nor is it known whether different interneuron subclasses show substantial resonance properties.

To bridge this gap, we combined Patch-seq data from human and mouse neurons obtained in both acute slice and organotypic culture preparations with conductance-based modelling to systematically investigate the mechanisms underlying resonance in human and mouse LAMP5, VIP, PV, and SST interneurons. Leveraging the triple modality patch-seq[25] dataset that combines transcriptomic, electrophysiological and morphological signatures of human interneuron subclasses, we address which resonance-related ion channel genes, conductances and morphological features drive subthreshold resonance and its prevalence in human interneurons. Finally, we build detailed computational models of human and mouse interneurons to systematically interrogate how and which of these mechanisms influence subthreshold resonance and contribute to frequency tuning in *in vivo*-like conditions.

### Results

#### Human interneurons show widespread and stronger resonance

Subthreshold resonance in interneurons is critical for brain function, enabling cells to respond preferentially to specific input frequencies, organize the precise timing of action potentials and synchronize neural networks during cognitive processes[18,26]. We first asked how adult human GABAergic interneurons respond to subthreshold resonant inputs compared with those in mice. To this end, we analyzed previously published Patch-seq datasets complemented by newly generated Patch-seq recordings of electrophysiological, morphological, and transcriptomic profiles of human and mouse cortical GABAergic interneurons[2,27] (**Fig. 1a**). In total, our dataset comprised 1085 human interneurons from 259 subjects (542 acute from 170 donors; 543 cultured from 89 donors; **Table S1**, **Fig. S1a-b**) and 619 acutely recorded mouse interneurons from 293 mice (**Fig. 1b, Table S2**).

To determine subthreshold resonant properties during a whole-cell patch-clamp recording, we measured acutely recorded interneuron responses across all cortical layers to a chirp stimulus – a constant-amplitude sinusoidal injection with increasing frequency from 0.4 to 40 Hz over 20 seconds. To quantify how a neuron filters different frequencies, we constructed an impedance profile (Z; **Fig. 1a**). Neurons can exhibit either a typical low-pass filtering response (the grey line in **Fig. 1a**, far-right plot) or a band-pass filtering response with a distinct peak in the impedance profile (the black line), indicative of resonance. The frequency at which this peak occurs ($Z_{max}$) was defined as peak resonance frequency ($f_{\mathrm{res}}$), and the strength of resonance ($Q$) was calculated as the ratio of the impedance at the peak resonance frequency ($Z_{max}$) to the impedance at the lowest frequency ($Z_0$; **Fig. 1a**). Interneurons were classified as resonant if they exhibited a noticeable peak ($Q > 1.05$[28]) in the impedance profile at a non-zero frequency above 1 Hz ($f_{\mathrm{res}} > 1$ Hz). The primary resonance metrics did not differ between cortical regions of surgical resection (frontal, temporal, parietal, or occipital; **Fig. S1c**, **e-i**) or donor's disease type (epilepsy vs tumor; **Fig. S1d**, **j-n**).

We found that both human and mouse interneurons exhibited resonance peaks roughly in the 2-5 Hz range (**Fig. 2a-b**). Human cortical interneurons had higher peak resonance frequencies ($f_{\mathrm{res}}$: $p < 0.001$), resonance strengths ($Q$: $p < 0.001$; Kolmogorov-Smirnov tests; **Fig. 2b**), and proportions of resonant cells ($f_{\mathrm{res}} > 1$Hz and

$Q > 1.05$) compared to mouse cortical interneurons (32.7% in human vs 9.3% in mouse; $\chi^2$ (1) = 42.76, $p < 0.001$; **Fig. 2c**). Examining differences in resonance properties between subclasses of human and mouse interneurons (Methods, Subclass assignment), we found that human LAMP5 ($p < 0.001$), VIP ($p < 0.001$), and PV interneurons ($p < 0.001$) were more often resonant than their mouse counterparts (**Fig. 2d**). In contrast, SST ($p = 0.544$) interneurons exhibited similar fractions of resonant cells between species (**Fig. 2d**). Human interneurons consistently exhibited higher resonance prevalence compared to mouse across a range of increasingly stringent resonance criteria ($f_{res}$> 1Hz and $Q > 1.1$ or 1.2; **Fig. S2**).

We next asked whether the resonance frequency differed across interneuron subclasses and species. A linear mixed-effects model revealed species differences in peak resonance frequency that were qualified by subclass ($p = 0.006$; **Fig. 2e**). Specifically, we found that human PV ($p = 0.003$) and VIP ($p < 0.001$) interneurons had significantly higher peak resonance frequencies than their mouse counterparts. On the other hand, human and mouse LAMP5 ($p = 0.133$) and SST ($p = 0.701$) interneurons did not differ in peak resonance frequency. Similarly, the species differences in 3dB cutoff frequency were subclass-specific ($p < 0.001$; **Fig. 2f**): Human LAMP5 ($p < 0.001$), PV ($p < 0.001$), and SST ($p = 0.020$) interneurons showed lower 3dB cutoffs compared to mouse counterparts, while human and mouse VIP interneurons showed similar 3dB cutoff distributions ($p = 0.427$). Additionally, human PV ($p < 0.001$), VIP ($p < 0.001$), and LAMP5 ($p < 0.001$), but not SST ($p = 0.419$), interneurons showed higher resonance strengths compared to mouse counterparts (**Fig. 2g**). Together, these results suggest that human cortical interneurons exhibit widespread, more pronounced resonance and narrower band filtering around the peak resonance frequency compared to mouse interneurons.

### Voltage sag ratio and input resistance relate to resonance properties in human interneurons

The contribution of $I_h$ to resonance can be revealed by a characteristic voltage “sag” during hyperpolarizing current injection – a sudden drop in membrane potential and subsequent depolarization due to $I_h$ (**Fig. 3a, b**). In addition, passive membrane properties such as input resistance might also contribute. To test which biophysical parameters of human interneurons could explain the resonance properties we observed, we first examined differences in membrane potential sag and input resistance across species and interneuron subclasses. We found that species differences in sag ratio ($p < 0.001$; **Fig. 3c**) and input resistance ($p < 0.001$; **Fig. 3d**) were subclass-specific. Sag ratios were higher in human LAMP5 ($p < 0.001$), VIP ($p < 0.001$), PV ($p < 0.001$), and SST ($p = 0.001$) interneurons compared to mouse counterparts. Furthermore, human LAMP5 ($p < 0.001$) and PV ($p < 0.001$) interneurons, but not VIP ($p = 0.312$) or SST ($p = 0.108$) interneurons, had higher input resistances compared to mouse counterparts.

To test whether the sag ratio and input resistance were associated with resonance properties, we included them as covariates in a linear mixed-effects model. We found that both sag ratio and input resistance were predictive of peak resonance frequency in a subclass-specific manner among human interneurons (Sag: $p = 0.035$, **Fig. 3e;** Input resistance: $p = 0.029$, **Fig. 3g**). Separate linear mixed-effects models per subclass showed positive relationships between sag ratio and peak resonance frequency in human LAMP5 ($\beta = 3.31$, $p < 0.001$), VIP ($\beta = 4.79$, p < 0.001), and PV ($\beta = 6.13$, $p < 0.001$) interneurons (**Fig. 3e**). Input resistance was negatively related to peak resonance frequency in human LAMP5 ($\beta = -0.002$, $p < 0.001$) and PV ($\beta = -0.006$, $p < 0.001$) interneurons (**Fig. 3g**). In contrast, Sag ratio was not predictive of peak resonance frequency among mouse interneurons ($p = .113$, **Fig. 3f**) and only input resistance was a significant predictor of peak resonance frequency, and this effect did not differ between subclasses (**Fig. 3h**).

Like peak resonance frequency, sag ratio was strongly predictive of resonance strength among human interneurons ($\beta = .47$, $p < 0.001$), and this effect did not differ between subclasses (**Fig. 3i**). Correspondingly, input resistance was negatively related to resonance strength among human interneurons ($\beta = -0.0001$, $p = 0.005$; **Fig. 3k**). In mouse interneurons, sag ratio was weakly related to resonance strength ($\beta = .018$, $p = 0.021$, **Fig. 3j**), and input resistance did not relate to resonance strength ($p = 0.820$, **Fig. 3l**). Taken together, these results show that sag properties and input resistance of human interneurons are strongly correlated with resonance features, especially in PV, VIP, and LAMP5 interneurons, while these correlations are mostly absent in mouse interneurons.

**Resonance dependence on cortical depth locations**

In human and non-human primate supragranular pyramidal neurons, resonance-related properties scale with distance from the pia, with deeper layer 3

glutamatergic pyramidal neurons showing stronger resonance and a higher sag ratio than superficial layer 2 neurons[19,21]. To test whether this relationship holds for human and mouse cortical interneurons, we examined whether resonance features depended on the cell-body location in cortical depth (**Fig. 4**). The cell body depths in our sample ranged from 22 to 2379 µm in the human cortex (**Fig. 4a**), and 12 to 881 µm in the mouse cortex (Fig. 4H), spanning all cortical layers. Among cells with available morphological reconstructions[2,27], we found that total dendritic length increased with distance from the pia ($\beta$ = 1.35, $p$ < 0.002, **Fig. 4b**).

In contrast to the observed increase in sag ratio with distance from pia in pyramidal neurons[19,21,29,30], sag ratio decreased with larger distance from pia in human LAMP5 ($p$ = 0.001), VIP ($p$ = 0.008), and SST ($p$ = 0.002) interneurons, whereas it did not change in PV ($p$ = .478) interneurons (**Fig. 4c**). An overall decrease in sag ratio with cortical depth was also evident in mouse cortical interneurons ($p$ < 0.001) and did not vary by subclass ($p$ = 0.185, **Fig. 4i**). Importantly, in contrast to human pyramidal neurons, the pia-soma distance in human interneurons did not correlate with input resistance, peak resonance, or 3 dB cutoff frequency (**Fig. 4d-g**), while resonance strength even decreased with distance from pia when all interneuron subclasses were taken together ($p$ = 0.031**, Fig. 4g**). Resonance strength and frequency were also unrelated to distance from the pia in a sub-selection of L2/L3 human cortical interneurons (Fig. S2). Similarly, none of the resonance parameters scaled with cortical depth in mouse cortical interneurons (all $p$ > .05, **Fig. 4k-m**). We conclude that, unlike human L2/L3 pyramidal neurons, human and mouse cortical interneurons do not display cortical depth-dependent resonance properties.

#### $I_h$ supports resonance in human interneurons

To mechanistically test the contribution of $I_h$ in resonance generation in human interneurons, we manipulated $I_h$ conductance in surgically resected human cortical tissue (**Fig. 5a**). We first blocked HCN channels with ZD7288 (10 µM) in seven human interneurons sampled across the cortical depth, targeted based on their firing profiles and cell body shapes (**Fig. S3**). We measured the sag ratio, input resistance, peak resonance frequency, and resonance strength in each recorded neuron. Blocking HCN channels increased input resistance ($p$ = 0.004; **Fig. 5c**) and eliminated voltage sag ($p$ = 0.004, **Fig. 5b**) as well as resonance ($f_{res}$: $p$ = 0.031; Q: p = 0.031; **Fig. 5d, e**) in human cortical interneurons. Although sag ratio and resonance strength values were generally lower in mouse cortical interneurons, HCN channel blockade reduced sag ratio, resonance strength and frequency measures (Fig. S3). Taken together, these results suggest that higher $I_h$ in human interneurons drives the occurrence of resonant cells, peak resonance frequency, and resonance strength.

Increased sag ratio has been observed in adult human interneurons in organotypic cultures of surgically resected human cortical brain tissue[2]. This is noteworthy since organotypic culturing affords viral targeting of acutely under-sampled cell types, such as SST interneurons. We tested whether increased sag ratio in cultured interneurons is paralleled by enhanced resonance properties (**Fig. 5f**). Indeed, we observed higher sag ratios in cultured neurons for LAMP5 and PV interneurons (LAMP5: $p < 0.001$; PV: $p < 0.001$). Input resistance did not differ between preparations ($p = 0.095$, **Fig. 5h**). We next asked whether proportions of resonant cells differed between acute and cultured interneurons. We found a higher proportion of resonant cells among interneurons in organotypic brain slice culture (60.8% in cultured vs 32.7% in acute, $p < 0.001$; **Fig. 5i**). This was true for LAMP5 ($p = 0.002$), PV ($p < 0.001$) and SST ($p < 0.001$) interneurons but not VIP interneurons, which showed equal proportions of resonant cells ($p = 0.100$). Moreover, an increase in peak resonance frequency (LAMP5: $p = 0.007$, PV: $p < 0.001$; **Fig. 5j**) and strength (LAMP5: $p = 0.002$, PV: $p < 0.001$; SST: $p = 0.023$; **Fig. 5k**) paralleled an increase in sag ratio in cultured tissue. On the other hand, the 3 dB cutoff frequency did not differ between acute and culture conditions (**Fig. 5l**). Taken together, these results suggest that an observed increase in sag ratio in cultured interneurons is paralleled by an increase in occurrence and prominence of resonance, especially among LAMP5, PV, and SST interneurons.

#### Voltage-dependent ion channel gene expression associates with resonance

Neuronal resonance arises from the combined activity of several specific voltage-dependent ion channels[18,26]. At hyperpolarized potentials, resonance is primarily driven by *HCN* channels (H-resonance, *HCN1–4*). Among these, the fast kinetics of *HCN1* subunits contribute most significantly to resonance, compared with the slower kinetics of HCN4 subunits[31]. Notably, *HCN1-4* subunits show differential expression levels in human and mouse single-cell RNA-expression datasets (**Fig. 6a**; Allen Brain Atlas; Allen Institute of Brain Science), with *HCN1* showing higher levels in human versus mouse interneurons. Additionally, *HCN1* modulator TRIP8b protein

RNA readout, *PEX5L,* levels are elevated in human interneurons (**Fig. 6a**).

Conversely, M-resonance emerges at depolarized potentials via *KCNQ* channels (*KCNQ2–5*), which exhibit low activation thresholds and minimal inactivation[32]. Additionally, T-type and P/Q-type voltage-gated calcium channels generate and amplify resonance in cortical and brainstem neurons[33–36]. While inductive currents generate resonance, regenerative currents, such as the persistent sodium current ($I_{NaP}$), mediated by voltage-gated sodium channels such as *SCN8A*, increase resonance strength[37].

To address the contribution of these ion channels to resonance, we determined whether human interneuron subclasses exhibit distinct expression patterns of these resonance-related ion channel genes. To this end, we used the Patch-seq transcriptomic data, which provide gene expression profiles for each recorded cell, enabling analysis of molecular covariates of electrophysiological features in the same cells. In both acute and cultured human cortical slices, all interneuron types showed high levels of expression of *HCN1*, *KCNQ*3/5, and *CACNA1A*/*G* gene transcripts (**Fig. 6b, Fig. S4a**). Other genes (*KCNJ16* and *CACNA1G* were expressed in most subclasses with lower mean expression levels. We next correlated the expression levels of these genes with resonance properties ($Q$, $f_{\text{res}}$) across interneurons collected from acute (**Fig. 6c**) and culture (**Fig. S4a**) preparations. After correction for multiple comparisons (FDR), we found significant positive relationships between *KCNJ16* and resonance strength ($n$ = 204, $r$ = 0.28, $p$ < 0.001) as well as resonance frequency ($n$ = 204, $r$ = 0.20, $p$ = 0.004) in the acute dataset. *HCN1* ($N$ = 240, $r$ = 0.17, $p$ = 0.007) and *CACNA1A* ($n$ = 254, $r$ = 0.16, $p$ = 0.009) also positively correlated to resonance frequency (**Fig. 6c, Table S3**). None of the correlations survived multiple correction in the cultured dataset (**Fig. S4a**). In contrast, *HCN2*, but none of the other genes was significantly related to resonance among mouse interneurons (**Fig. S4b**). Together, these findings suggest that in human interneurons with strong resonance properties, higher expression of the cyclic nucleotide-gated channel *HCN1* gene, Kir-type potassium channel gene *KCNJ16* and voltage-activated calcium channel gene *CACNA1A* may support membrane potential resonance properties.

**Large human interneurons require increased active properties for resonance**

To assess the contributions of voltage-gated ion channels enriched in human cortical interneurons to subthreshold resonance, we employed conductance-based computational interneuron models. We chose to model human PV interneurons specifically as they showed strong resonance properties (**Fig. 5**) and adapted our multi-compartmental model[3], featuring an average idealized human PV interneuron morphology, several active conductances ($Na^+$, $K^+$, and $I_h$) and a passive leak conductance (for exact parameter values, see Table S5). To investigate the effect of $I_h$ on resonance, the maximal $I_h$ density ($\overline{g}$) in the baseline model (+ $I_h$, pink trace) was removed (- $I_h$, purple trace) or doubled (+ $I_h$ *2, orange trace; **Fig. 7a**), resulting in an abolished or strengthened voltage sag and chirp response, respectively (**Fig. 7a, b**). Additionally, $I_h$ scaling affected resonance properties (**Fig. 7c-e**): The baseline model showed a detectable, albeit weak ($Q$ = 1.04), resonance peak in the impedance profile, which was absent in the - $I_h$ model (**Fig. 7e**). Increasing the baseline $I_h$ conductance (+ $I_h$ * 2, orange trace) increased the resonance frequency and strength ($Q$ = 1.09, **Fig. 7e**; **Fig. S5a-h**) and decreased input resistance (**Fig. S5a-c, e**). To characterize the effect of passive input resistance on resonance, we additionally varied the passive leak conductance in the baseline model and found an increased peak resonance frequency but not resonance strength (**Fig. S5i-n**). This finding was in line with previous research showing an inverse relationship between membrane resistivity and peak resonance frequency, with a simultaneous shunting of resonance strength[24]. Thus, the change in resonance strength with increasing $I_h$ could not be explained by passive input resistance change alone.

Next, we assessed the contribution of calcium channels to resonance by adding a voltage-gated M-type potassium channel or a low-threshold-activated calcium channel to the model[38]. Adding the calcium channel to the model led to the emergence of rebound spiking after hyperpolarization as well as to a considerable strengthening of the resonance profile (+$I_h$ +*Ca*, green trace, **Fig. 7b** and **c**). Both the M-type potassium and the calcium conductance increased resonance frequency ($f_{res}$ = 5.89, **Fig. 7d**) and resonance strength ($Q$ = 1.25, **Fig. 7e**), with little change in sag ratio (Sag $ratio_{+Ih}$ = 0.22; Sag $ratio_{+Ih+Ca}$ = 0.22) or input resistance ($IR_{+Ih}$ = 102.42; $IR_{+Ih+Ca}$ = 108.45). These results indicate that other ion channels can be responsible for strongly resonant profiles of human cortical interneurons observed in our experimental recordings.

Most human interneuron types have larger morphologies than their mouse counterparts[2,3,27]. We next asked how the cell morphology could affect membrane potential resonance. To this end, we used the reference (average morphology between human and mouse), human, and mouse PV interneuron morphologies[3] (See **Table S5**; **Fig. 7f**). The maximal active and passive conductances were kept equal across the three models and were the same as the baseline model used above[3] (**Table S5**). We found that the sag ratio and resonance strength increased as the morphology increased in size (**Fig. 7f, g, i**). In contrast, the 3 dB cutoff and resonance frequencies decreased (**Fig. 7h, j**) as the morphology approached the mouse model, with the human model showing a sharper resonance profile.

To test whether our findings could be attributed to total conductance scaling due to morphology - as larger human interneurons would have higher total conductance - we normalized the total conductances across models by scaling the maximal active conductance in proportion to the change in total area (Table S5). Consistent with our previous results, we observed a sharper peak in the impedance profile in the human model and a higher peak resonance frequency in the mouse model (**Fig. S5**). However, resonance strength was no longer higher in the human model compared to the mouse model. Together, these findings suggest that while active properties of the membrane could determine the strength of resonance, dendritic morphological features may strongly influence the bandwidth of subthreshold resonance.

**Human resonant morphologies show firing-level resonance**

Thus far, we have shown that human interneurons are proportionally more resonant and show stronger resonance than their mouse counterparts, but the functional relevance of this difference is unknown. Hence, we next asked whether subthreshold resonance in a PV model would translate to firing-level resonance (**Table S5**, **Fig. 8a**). We found that the resonant model (+ $I_h$ +$Ca^{2+}$) was generally more excitable (rheobase: I = 130 pA, **Fig. 7c**) than the non-resonant model (- $I_h$ -$Ca^{2+}$) (rheobase: I = 150 pA, **Fig. 8b**). While the non-resonant cell showed the highest excitability in response to the 1Hz stimulus (**Fig. 8b** (150 pA for 1Hz, 5 pA for 5 Hz), the resonant cell was most excitable in response to the 5 Hz stimulus (**Fig. 8c**; 130 pA for 5 Hz; 136 pA for 1 Hz), which corresponds approximately to its subthreshold resonance frequency (**Fig. 7d**).

In the *in vivo* brain, neuronal membrane potentials are highly dynamic due to ongoing barrages of synaptic inputs[39,40]. This raises the question whether resonant human interneurons would time their action potential firing to inputs arriving close to their preferred frequency. To this end, we injected sinusoidal currents of varying frequencies (1-20 Hz) together with in-vivo-like background noise current (see **Table S5** and **Fig. 8d**). Modelled neurons were slightly depolarized to generate action potentials at an average frequency of 10Hz. The timing of action potential generation was substantially modulated by the injected sinusoidal current hidden in the noise and showed a marked phase-dependence, which we quantified as the modulation index (M/R; **Fig. 8d, e**). M/R index quantifies how strongly a neuron's spiking is phase-locked to a periodic stimulus by taking the ratio of its peak modulation magnitude (M) to its average firing rate (R). The strongly resonating $+I_h$ $+Ca^{2+}$ model showed a peak in the normalized modulation index (**Fig. 8e**) and the signal gain functions[26] (**Fig. 8f**) which was absent in the $+I_h$ and $-I_h$ models. Additionally, while all three models showed comparable phases of signal gain at 1 Hz, only the strongly resonant model fired in phase with the 5 Hz stimulus (**Fig. 8g**). The same was true when baseline firing was normalized between models (**Fig. S6a-d**).

Finally, we tested whether the cell morphology could influence firing-level frequency preference. We injected sinusoidal currents of 1 to 50 Hz, along with the background noise (**Table S5**), and compared the resulting peristimulus histograms across resonant morphological models (**Fig. 8h**). All three resonant models fired in phase with the 5 Hz stimulus (**Fig. 8k**). However, the human interneuron morphology model showed the strongest response to this input, as reflected by the highest modulation index and signal gain amplitude at 5 Hz (**Fig. 8i-k**). While showing a clear delta-range peak in signal gain, the model of the human morphology failed to keep up with higher-frequency inputs (**Fig. 8h-k**). This steep drop in phase-locking (**Fig. 8k**) and response reliability was not observed in the mouse, or the reference morphology models (**Fig. 8i, j**). All in all, we conclude that the subthreshold resonance properties can translate into firing-level frequency preference in a morphology-dependent manner.

### Discussion

In this study, we provide the first systematic investigation of sub-threshold membrane potential resonance of adult human cortical interneuron types. By recording and analysing Patch-seq data from 1085 human interneurons and 619 mouse interneurons, we found that (1) in contrast to adult mouse interneurons, resonance is widespread and strong in human cortical interneurons, with a narrow bandwidth and a clear peak detected between 1 and 6 Hz. (2) Resonance is particularly prevalent in human PV interneurons. (3) Resonance properties of human interneurons are not cortical depth-dependent, and (4) are strongly related to hyperpolarization-activated voltage sag and input resistance. (5) HCN channel blockade eliminates resonance in human interneurons. (6) Human cortical interneurons have high levels of resonance-related gene expression, where *HCN1, CACNA1A and KCNJ16* transcripts are especially enriched in resonant interneurons. (7) Using conductance-based computational models with idealized human and mouse morphologies, we demonstrate that these voltage-dependent ion channels combined with morphological features afford human interneurons sharper frequency tuning which translates in the suprathreshold domain under noisy, in vivo-like conditions.

Our findings are in line with reports of delta-theta frequency band subthreshold resonance in human-specialized L1 interneurons[1] and putative L2/3-L5 cortical interneurons[30], suggesting that some interneurons in the human cortex are preferentially entrained by inputs arriving at these frequencies. Interestingly, the ranges of preferred frequencies largely overlap between subclasses and do not vary as a function of depth from pia, in contrast to human pyramidal neurons[19,21,29,30]. However, we observe a large spread of peak resonance frequencies within each subclass, likely reflecting variability in passive and active membrane properties among interneurons assigned to the same transcriptomic type and subclass identity[2]. Such heterogeneity might reflect finer cell-type distinctions[2].

Despite the observed variability within subclasses, we consistently found higher proportions of resonance in human LAMP5, VIP, and PV interneurons compared to their counterparts in the rodent brain. Species differences within the SST subclass are less pronounced and likely reflect limited SST sampling in human acute recordings. The proportion and strength of resonance was higher in SST neurons in organotypic adult human brain slice cultures, where virus-mediated EGFP expression in specific interneuron types aided SST neuron targeting leading to higher SST sample numbers[2].

Proportions and strengths of resonance were also higher in cultured PV and LAMP5 interneurons, which exhibit higher sag ratios compared to acutely recorded interneurons. Although, we show that general changes in input resistance are unlikely to be the cause, these differences could still arise from culturing-related changes in gene expression, dendritic remodeling, or network connectivity remodeling.

Both in acute as well as cultured human brain tissue, we found convergent correlational and experimental evidence that subthreshold resonance properties in human interneurons are largely $I_h$ -dependent, showing that the HCN-dependent mechanism of resonance observed in human cortical pyramidal neurons[19] generalises to the neighbouring inhibitory neurons. Additionally, we found $I_h$-dependence of resonance to be weaker in mouse interneurons, which is consistent with the generally weaker $I_h$-dependent subthreshold properties found in mouse cortical neurons found in this study, but as was also described by others[4,19]. Human interneurons displayed larger sag responses and a stronger contribution of HCN channels to membrane resistance, both of which suggest greater functional Ih than mouse. The enrichment of *HCN1* gene expression in human interneurons compared to the other slower subunits provides a plausible kinetic basis for subthreshold resonance, as the fast HCN1 channel activation is better suited to generating subthreshold resonance[20]. Previous immunohistochemistry examination has confirmed enriched HCN1 and HCN2 channels in human cortical fast-spiking interneurons compared to mouse[4]. The subcellular localisation of HCN channels may further explain the observed species difference in subthreshold resonance. Human fast-spiking interneurons show enriched somatic HCN expression, whereas rodent interneurons appear to show stronger axonal and weaker somatic expression[4,42,43]. Somatic enrichment would make HCN channels especially effective at shaping the voltage responses measured by somatic chirp stimulation. However, dendritic resonance may have not been reliably detected in our somatic recordings[17,44], therefore we take our data as evidence for stronger somatically detectable HCN-dependent resonance in human interneurons. Furthermore, differences in the expression of TRIP8b (the gene product of PEX5L) isoforms may contribute to the species differences in $I_h$-related properties we observed[45]. However, $I_h$ is unlikely to explain all the species-dependent differences in intrinsic properties we observed between mouse and human interneurons. Expression of voltage-gated calcium channel *CACNA1A* gene and inward rectifier potassium channel *KCNJ16* gene were associated with resonance strength and frequency of human interneurons.

A proposed role of $I_h$ in human interneurons is of a compensatory nature, evolved to overcome slow membrane kinetics inherent to large interneuron morphologies[4]. Our findings indirectly support this proposed role. In fact, given equal $I_h$ channel densities and kinetics, our computational models predicted that models with larger but less complex human-like morphological features would exhibit lower peak resonance and 3 dB cutoff frequencies and resonance strength than models with smaller but more complex mouse-like morphological features. In contrast scaling channel densities along with morphologies - such than human-like models carried a larger total $I_h$ density – resulted in predictions of stronger resonance responses among human-like morphological models. Irrespective of conductance scaling, however, computational models consistently predicted lower 3dB cutoffs in human-like morphological models, consistent with the sharp frequency tuning found in experimental recordings of human cortical interneurons.

Unlike human interneurons, mouse cortical interneurons exhibited largely flat and wide impedance profiles. However, a resonant peak was still detected in 9.3% of these interneurons, and this peak could be eliminated with HCN channel blockade. Notwithstanding some inconsistencies in definitions of resonance versus non-resonance, our findings are generally lower than the estimated 50% of resonance in cortical interneurons found in juvenile mice[33,46]. Other rodent studies find little to no resonance in FS interneurons in multiple brain areas[28,46,47], consistent with the near absence of HCN channel expression in mouse FS interneurons[4]. Thus, although other mechanisms have been proposed to generate subthreshold resonance in mouse interneurons[33], the evidence of $I_h$-dependent resonance was weak in mouse cortical interneurons at the membrane potentials sampled in this study.

**Limitations**

Our findings have a few limitations that are inherent to studying human brain tissue at this level of analysis. First, our computational insights rely on idealized morphologies. Future work using morphologically detailed, multi-compartment reconstructions will be essential to evaluate dendritic resonance and its specific contributions to local dendritic integration[3,17]. Second, while human tissue was obtained during resections for deep tumors or epilepsy—conditions linked to altered HCN channel expression—resonance properties did not correlate with patient pathology (Fig. S1l-n), giving confidence in their relevance. Finally, subthreshold resonance is state-dependent. While controlling for baseline membrane potential, our measurements likely represent conservative estimates of resonance capacity at more hyperpolarized potentials[22,47,48]. Moreover, peak resonance frequencies in the intact brain are likely higher due to elevated basal temperatures[49,50] and lower input resistance under high-conductance in vivo synaptic background[39].

**Functional implications**

The increased subthreshold resonance of human interneurons has suprathreshold implications. It was previously shown that low-frequency subthreshold resonance can be coupled to firing-rate resonance either within a single neuron, wherein highly noisy or low input amplitudes push the membrane voltage just over the firing threshold[18,26,51] or within *in vivo* networks, where theta-resonance is shown to emerge from $I_h$ -mediated post-inhibitory spiking in mice[14]. Here, we show that human neuron models with pronounced resonance profiles exhibit greater excitability around the peak subthreshold resonance frequency under noisy conditions in silico, likely mediating information transfer between distant, coincident networks[14]. Furthermore, the human morphology, more so than the mouse morphology, tunes to the theta frequency of inputs, possibly explaining how human interneurons tune to theta frequencies, as previously described in the human cortex *in vitro*[52].

The spiking activity of human cortical interneurons may be tuned to specific frequencies of synaptic input, and this selectivity may vary with interneuron type. The presence of subthreshold resonance in human, but not mouse, cortical interneurons, suggests a species difference in how inhibitory cells participate in ongoing network activity. Low-pass filtering properties in mouse interneurons indicate that they may primarily follow rhythms imposed by the circuit, whereas resonant human interneurons may bias the timing of inhibition within cortical rhythms[16,18,53]. Since interneurons form intricate connectivity patterns with pyramidal neurons, which are also tuned by resonance properties to specific input frequency patterns, human cortical networks are prone to generate activity in specific bandwidths, potentially contributing to the dominant delta/theta band oscillations apparent in human supragranular cortex[54]. The 2–6 Hz resonance observed here aligns with slow oscillations that peak in the cortex when cognitive control is required, such as upon surprising events or decision points, that involves integrating information from multiple brain regions[55]. This cognitive context is consistent with the broader idea that slow brain oscillations help coordinate neural activity across spatial and temporal scales[12]. Such rhythms can support long-range cortical communication through mechanisms including travelling waves, as well as through coordinating cross-frequency interactions, which are indeed strongly regulated by the rhythmic inhibition of cortical interneurons[52,56,57]. These converging observations may suggest that human cortical interneurons have adapted to the expanded human brain with stronger and widespread resonance properties to preserve temporal scaling of rhythmic oscillations.

### Methods and Materials

| REAGENT or RESOURCE | SOURCE | IDENTIFIER |
|---|---|---|
| Chemicals, peptides, and recombinant proteins | | |
| Alexa 488 | ThermoFisher | A10436 |
| Deposited data | | |
| | | |
| Experimental models: organisms/strains | | |
| Human neurosurgical samples | Table S1 | N/A |
| Mouse brain samples | Table S2 | C57Bl/6J |
| Software and algorithms | | |
| Python (v3.7.5) | Python Software Foundation | RRID:SCR_008394<br>https://www.python.org/downloads/release/python-3122/ |
| Anaconda | Anaconda Inc. | RRID:SCR_016142<br>https://www.anaconda.com/ |
| Spyder (v5.2.2) | Spyder Website | RRID:SCR_017585<br>https://www.spyder-ide.org |
| NumPy (v1.20.3) | Harris, C.R. et al, 2020<br>DOI:10.1038/s41586-020-2649-2 | RRID:SCR_008633<br>https://numpy.org/ |
| Pandas (v1.3.5) | Wes McKinney, 2010<br>doi.org/10.25080/Majora-92bf1922-00a | RRID:SCR_018214<br>https://pandas.pydata.org/ |
| Matplotlib (v3.4.2) | Hunter, 2007<br>doi.org/10.1109/MCSE.2007.55 | RRID:SCR_008624<br>https://matplotlib.org/ |
| Seaborn (v0.12.2) | Waskom, 2021<br>doi.org/10.21105/joss.03021 | RRID:SCR_018132<br>https://seaborn.pydata.org/ |
| IPFX (v2.0.0) | Allen Institute for Brain Science | N/A<br>https://github.com/AllenInstitute/ipfx |
| Scipy (v1.7.3) | Virtanen et al., (2020)<br>DOI: 10.1038/s41592-019-0686-2. | RRID:SCR_008058<br>https://scipy.org/ |
| Statsmodels (v0.13.2) | Seabold, S., & Perktold, J. (2025)<br>DOI: 10.25080/Majora-92bf1922-011 | RRID:SCR_016074<br>https://www.statsmodels.org/stable/index.html |
| Jupyter Notebooks (v7.0.8) | Kluyver, et al., 2016 | RRID: RRID:SCR_018413 |

| | | |
|---|---|---|
| | DOI: 10.25080/Majora-629e541a-00c | https://jupyter.org/ |
| NEURON (v8.2.6) | Hines & Carnevale, 2001 DOI: 10.1177/107385840100700207 | RRID:SCR_005393 https://www.neuronsimulator.org/en/9.0.0/guide/what_is_neuron.html |
| BluePyOpt (v1.14.18) | Van Geit et al., 2016 DOI: 10.3389/fninf.2016.00017 | RRID:SCR_014753 https://github.com/BlueBrain/BluePyOpt |
| NEURON model | Wilbers et al., 2023 (DOI: 10.1126/sciadv.adf0708) | PMID: 37824618 https://dataverse.nl/dataset.xhtml?persistentId=doi:10.34894/TONOHA |
| Igor Pro | | |
| MIES | | |
| Adobe Illustrator | Adobe Systems | RRID:SCR_010279 https://www.adobe.com/products/illustrator.html |
| SPSS (v29.0.2.0) | IBM Corp | RRID:SCR_002865 https://www.ibm.com/products/spss-statistics |
| Prism 10 | GraphPad Software | RRID:SCR_002798 https://www.graphpad.com/features |
| Neurolucida | MBF Bioscience | RRID:SCR_001775 https://www.mbfbioscience.com/neurolucida |
| Neurolucida Explorer | MBF Bioscience | RRID:SCR_017348 https://www.mbfbioscience.com/neurolucida-explorer |
| Author’s own code | Shamugia et al. | https://github.com/nsham2/Shamugia_et_al_2026/tree/main |
| Other | | |
| vibratome | Leica V1200S | N/A |
| Multi-Clamp 700B | Molecular Devices | N/A |

### Experimental Model and Study Participant Details

#### Human and mouse specimens

All ethical regulations on human and animal research were followed. Most of the human data were obtained from previously published manuscripts[2,27]. Newly generated human recordings (21 patients) were collected from apparently non-pathological neurosurgical tissue specimens collected during removal of deeper pathological tissue. This tissue was deemed non-essential for the diagnostic purposes and would have been discarded otherwise. All patients gave informed consent to the use of materials in this study. Tissue from 12 participants was obtained by the Allen Institute for Brain Science, and tissue from nine participants was obtained by the Vrije Universiteit Amsterdam with approval of the Vrije Universiteit Ethical Board. All patients had provided informed consent, and procedures were performed in accordance with the Declaration of Helsinki and with the approval of the VU Medical Centre Ethical Committee.

The data were collected from temporal, frontal, parietal, and occipital cortices (Fig. S2). Together, the study includes data from 224 donors aged 19-83 (Acute: $M_{age}$: 45.26 ± 1.64; Culture: $M_{age}$ = 50.45 ± 2.83; Fig. S1, Table S1) and 292 mice (129 female: $M_{age}$ P55.7 ± 6.7; 163 males: $M_{age}$ P54.2 ± 6.2; Table S2). Pathology type or brain area of origin did not consistently relate to resonance features (Fig. S2-3).

Additional patch-clamp mouse data was obtained from male ($n$ = 9) and female ($n$ = 9) juvenile (P4-6 weeks) wild-type C57Bl/6J mice. Whole brains were surgically removed following decapitation without the use of anaesthesia. All animals were housed < 5 per cage and had ad libitum access to food and water. Cages were maintained on a 12-hour light/dark cycle, at a temperature of 21.1°C and 45 to 70% humidity. All procedures were performed in accordance with the animal welfare guidelines and approval of the Vrije Universiteit Amsterdam Animal Ethical Committee.

### Method Details

#### Patch-seq datasets

See Lee, Dalley et al., 2023 and Chartrand et al., 2023 for detailed tissueprocessing methods. We processed and analysed the unpublished data in accordance with Lee, Dalley et al., 2023 and Chartrand et al., 2023.

*Subclass assignment*

The human interneuron subclass assignments (LAMP5/PAX6/Other (henceforth referred to as LAMP5), VIP, PV, SST subclasses) are largely based on the middle temporal gyrus (MTG) single-nucleus transcriptomic reference taxonomy[58], updated with insights from Patch-seq data[2]. For example, based on combined morphological, electrophysiological and transcriptomic data, the SST FRZB cell type is classified within the PV subclass. Mouse interneuron subclasses were either aligned to human subclasses based on their transcriptomic expression profile[27] or were assigned a subclass identity based on the mouse lines from which they were recorded (e.g., pv, sst cre-lines).

**Patch-clamp ZD7288 experiments**

Acute human surgically resected tissue was processed in accordance with the Patch-seq experimental workflow[25]. We targeted Interneurons across the cortical column and brain regions. Interneuron identity was determined upon visual inspection of Alexa Fluor 488-filled cells under the microscope and upon inspection of electrophysiological traces[59]. In the mouse cortex, interneurons were selectively targeted in the prefrontal cortex.

The experimental data was collected and saved in Igor software (Neurodata Without Borders (NWB) format) and analysed using IPFX python package and custom-built Python scripts. Voltage sag, input resistance, and resonance properties were measured at baseline (-65 mV) before and after a 10 µM ZD7288 wash-in.

To measure subthreshold membrane resonance, we injected the chirp stimulus - a sinusoidal current with a constant amplitude and a logarithmically increasing frequency of 0.4 Hz to 40 Hz over 20 seconds. The amplitude of the sinusoidal current injection was adjusted to produce a subthreshold membrane voltage response of $\pm$ 5 mV. The cell membrane potential was biased to -65 mV for the duration of the chirp recordings, unless otherwise specified.

Impedance amplitude profiles (ZAP) were derived by taking the ratio of the fast Fourier transform (FFT) of the voltage response (Vm) over the FFT of the injected current (Fig. 1A). The resulting impedance profile was smoothed (LOWESS) for further analysis. The peak resonance frequency ($f_{res}$) was defined as the frequency corresponding to peak impedance ($Z_{max}$). The 3 dB cutoff frequency was defined as the frequency at which the impedance reduced to 1/√2 of its maximum value. Resonance strength ($Q$) was defined as the ratio of maximal impedance over initial impedance ($Z_{max}$ /$Z_0$).

### Gene expression analyses

#### *RNA-seq*

RNA transcript levels of *HCN(1-4)* and *PEX5L* genes were obtained from a publicly available dataset (Allen Brain Map RNA-Seq; https://portal.brain-map.org/atlases-and-data/rnaseq). Specifically, single-nucleus SMART-seq data of all interneurons from human medial temporal gyrus (MTG; 2018), and mouse primary visual (V1) and anterior lateral motor cortices (ALM; 2018) were used for the analysis.

Linear mixed-effects models, with species as fixed effect and donor/animal as random intercept, were used to compare gene expression levels per gene (ln(CPM+1)) between human ($n$ = 4,164 from 8 donors) and mouse ($n$ = 10,534 from 210 mice) GABAergic interneurons. Both introns and exons were included in the analysis. Benjamini-Hochberg false discovery rate (FDR) correction was applied to account for multiple testing.

#### *Patch-seq*

Counts per million (CPM) expression values of a number of resonance-related genes (*"HCN1", "HCN2", "HCN3","HCN4", "KCNJ2", "KCNJ5, KCNJ11", "KCNJ16", "KCNQ2", "KCNQ3", "KCNQ5", "SCN1A", "SCN8A", "CACNA1A", "CACNA1H", "CACNA1G", "CACNA1I")* were obtained from acute and cultured human specimens. Per gene and subclass and preparation type, the proportion of expressing cells (CPM > 1.0) and mean expression levels were computed. Expression levels of a subset of prevalent genes were correlated with resonance parameters (Table S3). Benjamini–Hochberg false discovery rate (FDR) correction was applied per family of tests (acute and cultured datasets).

### Computational modelling

*FS model configurations*

Computational modelling was performed using NEURON[60] and BluePyOpt softwares[61]. A previously published FS neuron model[3] was adapted for subthreshold and suprathreshold analysis (Table S5). To study the effect of calcium and potassium channel conductances on resonance, calcium[38] and potassium[62] channels were inserted into to the model.

For morphological comparisons, three morphologies were applied (Table S5). To vary conductances, each maximal conductance ($\overline{g}$) was scaled accordingly.

*CHIRP Experiments*

Subthreshold resonance was examined using a long ($D$ = 62 seconds; dt = 0.1) chirp stimulus sweeping from 0.4 Hz ($f_{start}$) to 40 Hz ($f_{end}$) on a logarithmic time scale (c = log($f_{end}$ /$f_{start}$)):

$$\varphi(t) = 2\pi\left(\frac{f_{start}\cdot D}{c}\right)\left(e^{\frac{c}{D}t} - 1\right)$$

To avoid neuronal firing, the amplitude of injected current was auto calibrated such that the maximal initial deflection lay between ± 5 mV. The magnitude of impedance at any given frequency was calculated by dividing the fast-Fourier transform of voltage output by the fast-Fourier transform of current input:

$$Z = \frac{FFT(V)}{FFT(I)}$$

The resulting impedance profile was LOWESS smoothed (frac = 0.04) and the frequency corresponding to peak impedance ($Z_{max}$) was taken as peak resonance frequency ($f_{res}$), whereas the frequency corresponding to the 3 dB threshold ($Z_{max}/\sqrt{2}$) was taken as the 3 dB cutoff frequency. Resonance strength was defined as a ratio of peak and baseline impedances:

$$Q = \frac{Z_{min}}{Z_{max}}$$

*Modulation of firing*

To evaluate the modulation of firing at different frequencies of input, a sinusoid of amplitude $I$ and frequency $f$ was applied simultaneously with a DC baseline $I_0$ and randomly fluctuating noise based on[41]:

$$I(t) = I_0 + I_1 \sin(2\pi F t) + I_{noise}(t)$$

Exponentially filtered noise $I_{noise}(t)$ was defined as a zero-mean function with a standard deviation *sd* and correlation length of 5 msec sampled at 40 kHz.

$$I_{noise}(t + dt) = I_{noise}(t) \cdot \left(1 - \frac{dt}{\tau_I}\right) + sd \cdot \sqrt{\frac{2 \cdot dt}{\tau_I}} \cdot \xi_t$$

Where $\xi_t$ represents a sequence of pseudo-random numbers with a mean of 0 and standard deviation of 1. *Sd* was chosen to mimic a highly noisy synaptic background. Each sinusoidal current stimulus swept through 10 cycles, and the number of replicates (trials) per current injection were 10 * *f.* The exact parameter definitions per model are given in Table S5.

To estimate the firing rate modulation at any given frequency, trial-averaged instantaneous firing rates were computed for each bin (20 bins per cycle). This peri-stimulus time histogram (PSTH) was fitted with a sinusoidal function of the same frequency as the stimulation current. Modulation index was calculated as the ratio between the peak of the fitted sine function and the baseline firing (offset of the fitted sine). And the phase of signal gain ($\varphi$ ) was calculated as the delay of the sine function fit.

Amplitude of signal gain is taken as a firing-domain equivalent of neuronal frequency preference[24] and is defined as a ratio between peak frequency of firing and the magnitude of input current.

**Quantification and Statistical Analysis**

Statistical tests were conducted on SPSS (IBM) and Python software. For all statistical models and tests, see Table S3.

*Kolmogorov-Smirnov* tests were used to compare the distributions of peak resonance frequency and resonance strength between human and mouse interneurons.

*Chi-square* tests were used to compare the proportions of resonant cells ($F_{res} > 1$, $Q > 1.05$) between acute human and mouse interneurons or human acute and cultured interneurons. The expected counts in human acute contingency tables were based on the observed counts in mouse interneurons, and expected counts in human cultured contingency tables were based on human acute observed counts.

*Linear mixed-effects models*: Due to clustering in the data, where repeated measurements were collected from single donors, the data was fitted with a linear mixed-effects model with a random intercept for donor ID. Species, subclass, and their interaction were entered as fixed factors to evaluate the differences in subthreshold resonance properties. To account for the variability in these properties due to baseline membrane potential differences (Vm), this variable was included as a continuous covariate. Type III Wald F-tests with Satterthwaite's approximation was used to assess the main and interaction effects. Due to the deviations from Normality observed in Q-Q plots of residuals, the results from mixed linear models were confirmed using generalized estimating equations which do not rely on the assumption of normal residuals. Post-hoc contrasts between species on each level of subclass were conducted using Mann-Whitney U tests to account for non-Normality of data.

To evaluate the predictive power of active and passive membrane properties, subthreshold resonance estimates ($f_{res}$, $Q$) were fitted with a mixed linear model with donor ID entered as a random intercept to account for clustering in the data. Sag ratio and input resistance were entered as continuous covariates. Subclass identity as well as subclass ~ sag ratio and subclass ~ input resistance interactions were entered as fixed factors. Due to the deviations from Normality observed in Q-Q plots of residuals, the results from mixed linear models were confirmed using generalized estimating equations which do not rely on the assumption of normal residuals. To follow up on significant interaction effects, we ran linear mixed-effects models on each level of subclass with sag ratio and input resistance as continuous covariates as donor ID as random intercept. The same approach was used to establish the relationships between pia-soma distance and passive membrane properties of interest.

*Wilcoxon signed-rank tests*: To analyze within-interneuron ZD7288 experiments, we performed Wilcoxon signed-rank tests for testing two non-parametric paired groups.

### Author Contributions

**Natia Shamugia**: Conceptualization, Methodology, Software, Formal analysis, Investigation, Writing – Original Draft, Writing – Review & Editing, Visualization, Project administration. **Robin Weiler**: Conceptualization, Software, Writing – Original Draft. **Tim Heistek**: Investigation, Resources. **Kamiliam Nasirova**: Resources, Data Curation. **Sarah Scott**: Software, Formal Analysis, Investigation, Writing – Original Draft. **Sara Vargas**: Formal analysis, Investigation, Visualization. **Brian Lee**: Conceptualization, Methodology, Investigation, Resources, Data Curation, Writing – Original Draft. **Rachel Dalley**: Conceptualization, Methodology, Investigation, Resources, Data Curation, Writing – Original Draft. **Brian Kalmbach**: Conceptualization, Methodology, Writing – Original Draft**. Jonathan Ting**: Conceptualization, Methodology, Resources. **Ed S. Lein**: Funding acquisition. **Natalia Goriounova:** Conceptualization, Methodology, Formal analysis, Writing – Original Draft, Supervision, Funding acquisition. **Huibert D. Mansvelder**: Conceptualization, Methodology, Formal analysis, Writing – Original Draft, Supervision, Funding acquisition

### Funding

This project has received granting from European Research Council advanced grant "Fasthumanneuron" number 101093198 (H.D.M.); the NWO Gravitation program BRAINSCAPES: A Roadmap from Neurogenetics to Neurobiology (NWO: grant number 024.004.012) (H.D.M.); from the European Research Council (ERC) under the European Union's Horizon Europe research and innovation programme ERC consolidator grant "HumanCircuits" number 101229280 (N.A.G.); Dutch Research Council (NWO) under NWO-Talentprogramma 2021 VIDI grant number VI.Vidi.213.014 (N.A.G.); from Dutch Research Council (NWO) under the research programme 'NWO Open Competition Domain Science – XL' grant "SuperGlue" number OCENW.XL.23.041 (N.A.G.); National Institutes of Health (NIH) research grant UM1 MH130981 (E.S.L.).

### Competing interests

The authors declare no competing interests.

### Additional information

**Supplemental information:** The online version will contain supplemental material.

**Correspondence**: Requests for further information should be directed to Huibert Mansvelder (h.d.mansvelder@vu.nl) and Natalia Goriounova (n.a.goriounova@vu.nl).

**Open Access data and code Availability:** Original code will be made available at https://github.com/nsham2/Shamugia_et_al_2026. Most of the data used in this paper is already open source2,27. The remaining data will be made available upon request.

## References

1. Boldog, E., Bakken, T.E., Hodge, R.D., Novotny, M., Aevermann, B.D., Baka, J., Bordé, S., Close, J.L., Diez-Fuertes, F., Ding, S.L., et al. (2018). Transcriptomic and morphophysiological evidence for a specialized human cortical GABAergic cell type. Nature Neuroscience *21*, 1185–1195. https://doi.org/10.1038/s41593-018-0205-2.

2. Lee, B.R., Dalley, R., Miller, J.A., Chartrand, T., Close, J., Mann, R., Mukora, A., Ng, L., Alfiler, L., Baker, K., et al. (2023). Signature morphoelectric properties of diverse GABAergic interneurons in the human neocortex. Science *382*. https://doi.org/10.1126/science.adf6484.

3. Wilbers, R., Galakhova, A.A., Driessens, S.L., Heistek, T.S., Metodieva, V.D., Hagemann, J., Heyer, D.B., Mertens, E.J., Deng, S., Idema, S., et al. (2023). Structural and functional specializations of human fast-spiking neurons support fast cortical signaling. Science Advances *9*. https://doi.org/https://doi.org/10.1126/sciadv.adf0708.

4. Szegedi, V., Bakos, E., Furdan, S., Kovács, B.H., Varga, D., Erdélyi, M., Barzó, P., Szücs, A., Tamás, G., and Lamsa, K. (2023). HCN channels at the cell soma ensure the rapid electrical reactivity of fast-spiking interneurons in human neocortex. PLoS Biology *21*. https://doi.org/10.1371/journal.pbio.3002001.

5. Fang, R., Xia, C., Close, J.L., Zhang, M., He, J., Huang, Z., Halpern, A.R., Long, B., Miller, J.A., Lein, E.S., et al. (2022). Conservation and divergence of cortical cell organization in human and mouse revealed by MERFISH. Science *377*. https://doi.org/10.1126/science.abm1741.

6. Johansen, N., Somasundaram, S., Travaglini, K.J., Yanny, A.M., Shumyatcher, M., Casper, T., Cobbs, C., Dee, N., Ellenbogen, R., Ferreira, M., et al. (2023). Interindividual variation in human cortical cell type abundance and expression. Science *382*. https://doi.org/10.1126/science.adf2359.

7. Loomba, S., Straehle, J., Gangadharan, V., Heike, N., Khalifa, A., Motta, A., Ju, N., Sievers, M., Gempt, J., Meyer, H.S., et al. (2022). Connectomic comparison of mouse and human cortex. Science *377*. https://doi.org/10.1126/science.abo0924.

8. Molnár, G., Oláh, S., Komlósi, G., Füle, M., Szabadics, J., Varga, C., Barzó, P., and Tamás, G. (2008). Complex Events Initiated by Individual Spikes in the Human Cerebral Cortex. PLoS Biol *6*. https://doi.org/10.1371/journal.pbio.0060222.g001.

9. Campagnola, L., Seeman, S.C., Chartrand, T., Kim, L., Hoggarth, A., Gamlin, C., Ito, S., Trinh, J., Davoudian, P., Radaelli, C., et al. (2022). Local connectivity and synaptic dynamics in mouse and human neocortex. Science *375*. https://doi.org/10.1126/science.abj5861.

10. Molnár, G., Rózsa, M., Baka, J., Holderith, N., Barzó, P., Nusser, Z., and Tamás, G. (2016). Human pyramidal to interneuron synapses are mediated by multi-vesicular release and multiple docked vesicles. eLife *5*. https://doi.org/10.7554/eLife.18167.001.

11. Hunt, S., Leibner, Y., Mertens, E.J., Barros-Zulaica, N., Kanari, L., Heistek, T.S., Karnani, M.M., Aardse, R., Wilbers, R., Heyer, D.B., et al. (2023). Strong and reliable synaptic communication between pyramidal neurons in adult human cerebral cortex. Cerebral Cortex *33*, 2857–2878. https://doi.org/10.1093/cercor/bhac246.

12. Buzsáki, G., Logothetis, N., and Singer, W. (2013). Scaling brain size, keeping timing: Evolutionary preservation of brain rhythms. Neuron *80*, 751–764. https://doi.org/10.1016/j.neuron.2013.10.002.

13. Lampl, I., and Yarom, Y. (1997). Subthreshold oscillations and resonant behavior: two manifestations of the same mechanism. Neuroscience *78*, 325–341.

14. Stark, E., Eichler, R., Roux, L., Fujisawa, S., Rotstein, H.G., and Buzsáki, G. (2013). Inhibition-Induced theta resonance in cortical circuits. Neuron *80*, 1263–1276. https://doi.org/10.1016/j.neuron.2013.09.033.

15. Stark, E., Levi, A., and Rotstein, H.G. (2022). Network resonance can be generated independently at distinct levels of neuronal organization. PLoS Computational Biology *18*. https://doi.org/10.1371/journal.pcbi.1010364.

16. Izhikevich, E.M. (2001). Resonate-and-fire neurons. Neural Netw. *14*, 883–894. https://doi.org/doi:10.1016/s0893-6080(01)00078-8.

17. Narayanan, R., and Johnston, D. (2007). Long-Term Potentiation in Rat Hippocampal Neurons Is Accompanied by Spatially Widespread Changes in Intrinsic Oscillatory Dynamics and Excitability. Neuron *56*, 1061–1075. https://doi.org/10.1016/j.neuron.2007.10.033.

18. Hutcheon, B., and Yarom, Y. (2000). Resonance, oscillation and the intrinsic frequency preferences of neurons. Trends Neurosci. *23*, 216–222. https://doi.org/doi:10.1016/s0166-2236(00)01547-2.

19. Kalmbach, B.E., Buchin, A., Long, B., Close, J., Nandi, A., Miller, J.A., Bakken, T.E., Hodge, R.D., Chong, P., de Frates, R., et al. (2018). h-Channels Contribute to Divergent Intrinsic Membrane Properties of Supragranular Pyramidal Neurons in Human versus Mouse Cerebral Cortex. Neuron *100*, 1194-1208.e5. https://doi.org/10.1016/j.neuron.2018.10.012.

20. Rich, S., Moradi Chameh, H., Sekulic, V., Valiante, T.A., and Skinner, F.K. (2021). Modeling Reveals Human-Rodent Differences in H-Current Kinetics Influencing Resonance in Cortical Layer 5 Neurons. Cerebral Cortex *31*, 845–872. https://doi.org/10.1093/cercor/bhaa261.

21. Radaelli, C., Schmitz, M., Liu, X.-P., Sawchuk, S., Opitz-Araya, X., Hudson, M., Taskin, N., Bertagnolli, D., Goldy, J., Ko, A.L., et al. (2026). HCN channels reveal conserved and divergent physiology in supragranular pyramidal neurons in primate species. Communications Biology *9*. https://doi.org/10.1038/s42003-026-09558-2.

22. Robinson, R.B., and Siegelbaum, S.A. (2003). Hyperpolarization-Activated Cation Currents: From Molecules to Physiological Function. Annual Review of

Physiology *65*, 453–480. https://doi.org/10.1146/annurev.physiol.65.092101.142734.

23. Nolan, M.F., Malleret, G., Dudman, J.T., Buhl, D.L., Santoro, B., Gibbs, E., Vronskaya, S., Buzsáki, G., Siegelbaum, S.A., Kandel, E.R., et al. (2004). A behavioral role for dendritic integration: HCN1 channels constrain spatial memory and plasticity at inputs to distal dendrites of CA1 pyramidal neurons. Cell *119*, 719–732. https://doi.org/10.1016/j.cell.2004.11.020.

24. Narayanan, R., and Johnston, D. (2008). The h channel mediates location dependence and plasticity of intrinsic phase response in rat hippocampal neurons. Journal of Neuroscience *28*, 5846–5860. https://doi.org/10.1523/JNEUROSCI.0835-08.2008.

25. Lee, B.R., Budzillo, A., Hadley, K., Miller, J.A., Jarsky, T., Baker, K., Hill, D., Kim, L., Mann, R., Ng, L., et al. (2021). Scaled, high fidelity electrophysiological, morphological, and transcriptomic cell characterization. eLife. https://doi.org/10.7554/eLife.

26. E Richardson, M.J., Brunel, N., Hakim, V., and E, M.J. (2003). From Subthreshold to Firing-Rate Resonance. J Neurophysiol *89*, 2538–2554. https://doi.org/10.1152/jn.00955.

27. Chartrand, T. (2023). Morpho-electric and transcriptomic divergence of the layer 1 interneuron repertoire in human versus mouse neocortex. Science. https://doi.org/10.48324/dandi.000630/0.230915.2257.

28. Zemankovics, R., Káli, S., Paulsen, O., Freund, T.F., and Hájos, N. (2010). Differences in subthreshold resonance of hippocampal pyramidal cells and interneurons: The role of h-current and passive membrane characteristics. Journal of Physiology *588*, 2109–2132. https://doi.org/10.1113/jphysiol.2009.185975.

29. Dalley, R., Walling-Bell, S., Miller, J.A., Waleboer, F., Thijssen, J., McCutcheon, A., Gelfand, E., Mallory, M., Radaelli, C., Liu, X.-P., et al. (2026). Human

Neocortical Glutamatergic Neurons Revealed Through Multimodal Profiling. bioRxiv, 2026.01.08.698257. https://doi.org/10.64898/2026.01.08.698257.

30. Moradi Chameh, H., Rich, S., Wang, L., Chen, F.D., Zhang, L., Carlen, P.L., Tripathy, S.J., and Valiante, T.A. (2021). Diversity amongst human cortical pyramidal neurons revealed via their sag currents and frequency preferences. Nature Communications *12*. https://doi.org/10.1038/s41467-021-22741-9.

31. Mishra, P., and Narayanan, R. (2025). The enigmatic HCN channels: A cellular neurophysiology perspective. Proteins *93*, 72–92. https://doi.org/10.1002/prot.26643.

32. Eguchi, Y., Kuwano, Y., Okada, S., Morino, H., and Hashimoto, K. (2025). Kcnq (Kv7) channels exhibit frequency-dependent responses via partial inductor-like gating dynamics. Communications Biology *8*. https://doi.org/10.1038/s42003-025-08302-6.

33. Sun, H., An, S., Luhmann, H.J., and Kilb, W. (2014). Resonance properties of GABAergic interneurons in immature GAD67-GFP mouse neocortex. Brain Research *1548*, 1–11. https://doi.org/10.1016/j.brainres.2013.12.032.

34. Sun, H., Luhmann, H.J., and Kilb, W. (2012). Resonance properties of different neuronal populations in the immature mouse neocortex. European Journal of Neuroscience *36*, 2753–2762. https://doi.org/10.1111/j.1460-9568.2012.08196.x.

35. Hashimoto, K. (2020). Mechanisms for the resonant property in rodent neurons. Neuroscience Research *156*, 5–13. https://doi.org/10.1016/j.neures.2019.12.013.

36. Choi, S., Yu, E., Kim, D., Urbano, F.J., Makarenko, V., Shin, H.S., and Llinás, R.R. (2010). Subthreshold membrane potential oscillations in inferior olive neurons are dynamically regulated by P/Q- and T-type calcium channels: A study in mutant mice. Journal of Physiology *588*, 3031–3043. https://doi.org/10.1113/jphysiol.2009.184705.

37. Wengert, E.R., Miralles, R.M., Wedgwood, K.C.A., Wagley, P.K., Strohm, S.M., Panchal, P.S., Idrissi, A.M., Wenker, I.C., Thompson, J.A., Gaykema, R.P., et al. (2021). Somatostatin-Positive Interneurons Contribute to Seizures in SCN8A

Epileptic Encephalopathy. Journal of Neuroscience *41*, 9257–9273. https://doi.org/10.1523/JNEUROSCI.0718-21.2021.

38. McRory, J.E., Santi, C.M., Hamming, K.S.C., Mezeyova, J., Sutton, K.G., Baillie, D.L., Stea, A., and Snutch, T.P. (2001). Molecular and Functional Characterization of a Family of Rat Brain T-type Calcium Channels. Journal of Biological Chemistry *276*, 3999–4011. https://doi.org/10.1074/jbc.M008215200.

39. Destexhe, A., Rudolph, M., and Paré, D. (2003). The high-conductance state of neocortical neurons in vivo. Nature Reviews Neuroscience *4*, 739–751. https://doi.org/10.1038/nrn1198.

40. Gentet, L.J., Kremer, Y., Taniguchi, H., Huang, Z.J., Staiger, J.F., and Petersen, C.C.H. (2012). Unique functional properties of somatostatin-expressing GABAergic neurons in mouse barrel cortex. Nature Neuroscience *15*, 607–612. https://doi.org/10.1038/nn.3051.

41. Testa-Silva, G., Verhoog, M.B., Linaro, D., de Kock, C.P.J., Baayen, J.C., Meredith, R.M., De Zeeuw, C.I., Giugliano, M., and Mansvelder, H.D. (2014). High Bandwidth Synaptic Communication and Frequency Tracking in Human Neocortex. PLoS Biology *12*. https://doi.org/10.1371/journal.pbio.1002007.

42. Povysheva, N.V., Zaitsev, A.V., Rotaru, D.C., Gonzalez-Burgos, G., Lewis, D.A., and Krimer, L.S. (2008). Parvalbumin-positive basket interneurons in monkey and rat prefrontal cortex. Journal of Neurophysiology *100*, 2348–2360. https://doi.org/10.1152/jn.90396.2008.

43. Yang, S.S., Li, Y.C., Coley, A.A., Chamberlin, L.A., Yu, P., and Gao, W.J. (2018). Cell-type specific development of the hyperpolarization-activated current, Ih, in prefrontal cortical neurons. Frontiers in Synaptic Neuroscience *10*. https://doi.org/10.3389/fnsyn.2018.00007.

44. Zhuchkova, E., Remme, M.W.H., and Schreiber, S. (2013). Somatic versus dendritic resonance: Differential filtering of inputs through non-uniform distributions of active conductances. PLoS ONE *8*. https://doi.org/10.1371/journal.pone.0078908.

45. Lewis, A.S., Schwartz, E., Chan, C.S., Noam, Y., Shin, M., Wadman, W.J., Surmeier, D.J., Baram, T.Z., Macdonald, R.L., and Chetkovich, D.M. (2009). Alternatively spliced isoforms of TRIP8b differentially control h channel trafficking and function. Journal of Neuroscience *29*, 6250–6265. https://doi.org/10.1523/JNEUROSCI.0856-09.2009.

46. Binini, N., Talpo, F., Spaiardi, P., Maniezzi, C., Pedrazzoli, M., Raffin, F., Mattiello, N., Castagno, A.N., Masetto, S., Yanagawa, Y., et al. (2021). Membrane Resonance in Pyramidal and GABAergic Neurons of the Mouse Perirhinal Cortex. Frontiers in Cellular Neuroscience *15*. https://doi.org/10.3389/fncel.2021.703407.

47. Hutcheon, B., Miura, R.M., and Puil, E. (1996). Subthreshold Membrane Resonance in Neocortical Neurons.

48. Dwyer, J., Lee, H., Martell, A., and van Drongelen, W. (2012). Resonance in neocortical neurons and networks. European Journal of Neuroscience *36*, 3698–3708. https://doi.org/10.1111/ejn.12001.

49. Rzechorzek, N.M., Thrippleton, M.J., Chappell, F.M., Mair, G., Ercole, A., Cabeleira, M., Rhodes, J., Marshall, I., and O'neill, J.S. (2022). A daily temperature rhythm in the human brain predicts survival after brain injury. Brain *145*, 2031–2048. https://doi.org/10.1093/brain/awab466.

50. Hu, H., Vervaeke, K., and Storm, J.F. (2002). Two forms of electrical resonance at theta frequencies, generated by M-current, h-current and persistent Na+ current in rat hippocampal pyramidal cells. Journal of Physiology *545*, 783–805. https://doi.org/10.1113/jphysiol.2002.029249.

51. Rotstein, H.G. (2017). Spiking resonances in models with the same slow resonant and fast amplifying currents but different subthreshold dynamic properties. Journal of Computational Neuroscience *43*, 243–271. https://doi.org/10.1007/s10827-017-0661-9.

52. Florez, C.M., McGinn, R.J., Lukankin, V., Marwa, I., Sugumar, S., Dian, J., Hazrati, L.N., Carlen, P.L., Zhang, L., and Valiante, T.A. (2015). In vitro recordings of human neocortical oscillations. Cerebral Cortex *25*, 578–597. https://doi.org/10.1093/cercor/bht235.

53. Buzsáki, G., and Draguhn, A. (2004). Neuronal Oscillations in Cortical Networks. Science *304*. https://doi.org/doi:10.1126/science.1099745.

54. Halgren, M., Fabó, D., Ulbert, I., Madsen, J.R., Eross, L., Doyle, W.K., Devinsky, O., Schomer, D., Cash, S.S., and Halgren, E. (2018). Superficial Slow Rhythms Integrate Cortical Processing in Humans. Scientific Reports *8*. https://doi.org/10.1038/s41598-018-20662-0.

55. Boroujeni, K.B., Balaram, P., Tiesinga, P., and Womelsdorf, T. (2026). Prefrontal cortex interneurons and their contributions to attention, working memory, and adaptive behavior. Progress in Neurobiology *258*. https://doi.org/10.1016/j.pneurobio.2025.102873.

56. Zhang, H., Watrous, A.J., Patel, A., and Jacobs, J. (2018). Theta and Alpha Oscillations Are Traveling Waves in the Human Neocortex. Neuron *98*, 1269-1281.e4. https://doi.org/10.1016/j.neuron.2018.05.019.

57. Debnath, R., Elyamany, O., Iffland, J.R., Rauh, J., Siebert, M., Andraes, E., Leicht, G., and Mulert, C. (2025). Theta transcranial alternating current stimulation over the prefrontal cortex enhances theta power and working memory performance. Front. Psychiatry *15*, 1493675. https://doi.org/10.3389/fpsyt.2024.1493675.

58. Hodge, R.D., Bakken, T.E., Miller, J.A., Smith, K.A., Barkan, E.R., Graybuck, L.T., Close, J.L., Long, B., Johansen, N., Penn, O., et al. (2019). Conserved cell types with divergent features in human versus mouse cortex. Nature *573*, 61–68. https://doi.org/10.1038/s41586-019-1506-7.

59. Defelipe, J., López-Cruz, P.L., Benavides-Piccione, R., Bielza, C., Larrañaga, P., Anderson, S., Burkhalter, A., Cauli, B., Fairén, A., Feldmeyer, D., et al. (2013). New insights into the classification and nomenclature of cortical GABAergic interneurons. Nature Reviews Neuroscience *14*, 202–216. https://doi.org/10.1038/nrn3444.

60. Hines, M.L., and Carnevale, N.T. (2001). NEURON: a tool for neuroscientists. THE NEUROSCIENTIST *7*. https://doi.org/doi:10.1177/107385840100700207.

61. Van Geit, W., Gevaert, M., Chindemi, G., Rössert, C., Courcol, J.D., Muller, E.B., Schürmann, F., Segev, I., and Markram, H. (2016). BluePyOpt: Leveraging open source software and cloud infrastructure to optimise model parameters in neuroscience. Frontiers in Neuroinformatics *10*. https://doi.org/10.3389/fninf.2016.00017.

62. Bhalla, U.S., and Bower, J.M. (1993). Exploring Parameter Space in Detailed Single Neuron Models: Simulations of the Mitral and Granule Cells of the Olfactory Bulb.

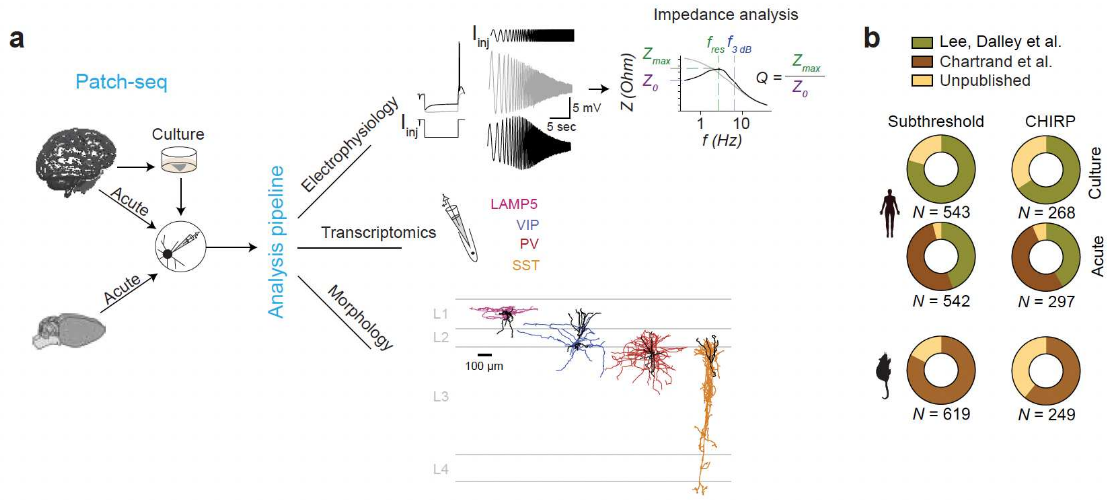

**Fig. 1 | Patch-seq data description.**

**a** Experimental workflow. Patch-seq recordings were obtained from human (acute and cultured) and mouse (acute) cortices for subthreshold and CHIRP analysis. From an averaged CHIRP sweep, an impedance profile was derived to calculate peak resonance frequency ($f_{res}$), 3 dB cutoff frequency ($f_{3\ dB}$) and resonance strength ($Q = Z_{max}/Z_0$). Transcriptomic profiling and gene expression data was obtained by RNA sequencing the nuclei of the patched cells.

**b** Cell counts *(n)* of published and newly generated data by species (human, mouse) and preparation (acute, cultured).

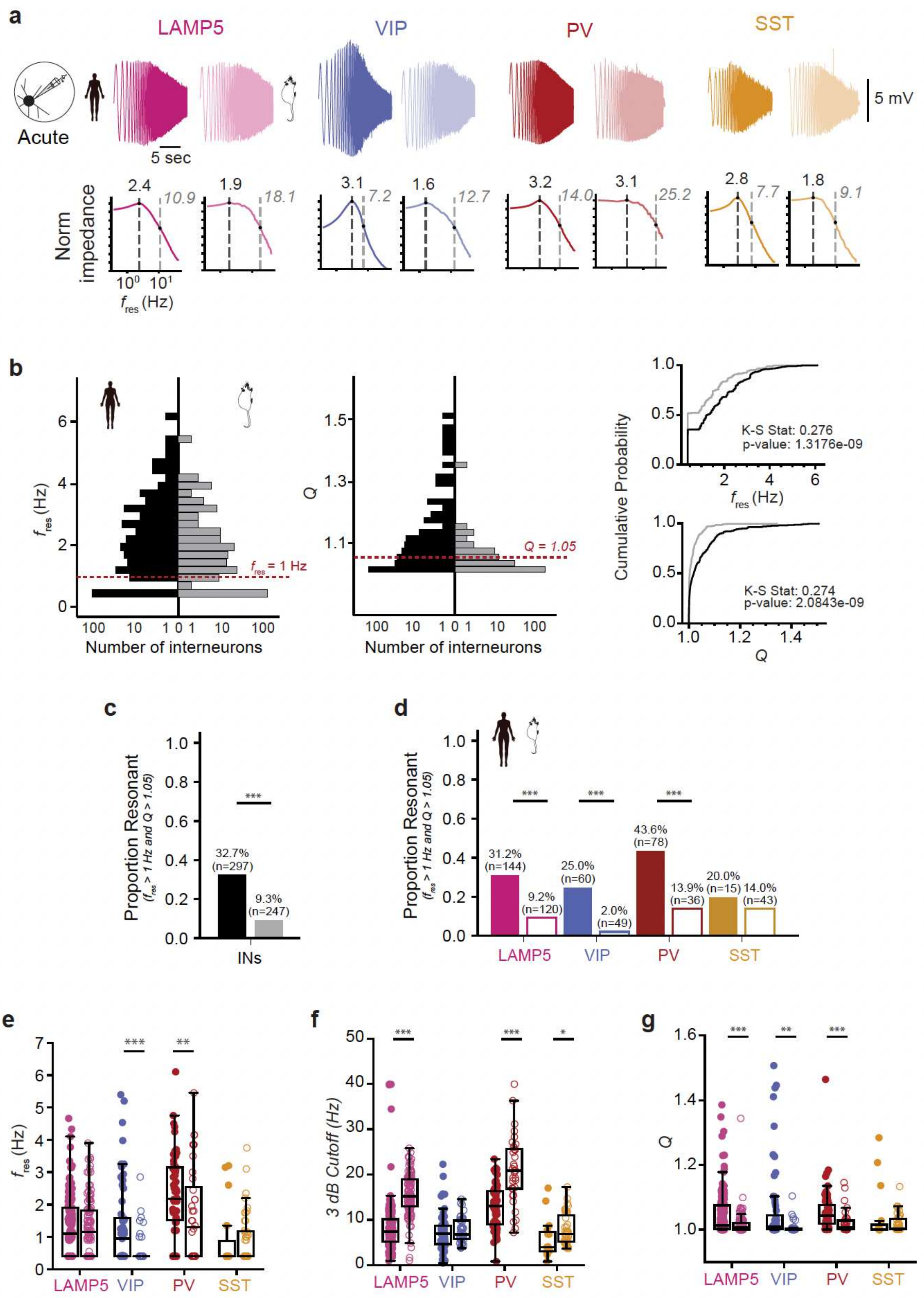

a
LAMP5
VIP
PV
SST
Acute
5 sec
5 mV
Norm impedance
$f_{res}$ (Hz)
2.4
10.9
1.9
18.1
3.1
7.2
1.6
12.7
3.2
14.0
3.1
25.2
2.8
7.7
1.8
9.1
b
$f_{res}$ (Hz)
$f_{res}$ = 1 Hz
Number of interneurons
Q
Q = 1.05
Cumulative Probability
K-S Stat: 0.276
p-value: 1.3176e-09
K-S Stat: 0.274
p-value: 2.0843e-09
c
Proportion Resonant ($f_{res}$ > 1 Hz and Q > 1.05)
32.7% (n=297)
9.3% (n=247)
INs
d
31.2% (n=144)
9.2% (n=120)
25.0% (n=60)
2.0% (n=49)
43.6% (n=78)
13.9% (n=36)
20.0% (n=15)
14.0% (n=43)
e
f
3 dB Cutoff (Hz)
g

**Fig. 2 | A high proportion of resonant cells among human PV interneurons.**

**a** Representative CHIRP voltage responses (top) and impedance profiles (bottom) by subclass and species. Dashed lines: $f_{res}$ (dark) and $f_{3\ dB}$ (light).

**b** $f_{res}$ (left) and $Q$ (middle) distributions for human ($n$ = 297) and mouse ($n$ = 268) interneurons, with corresponding cumulative probability plots (right; K-S test). Dashed lines: resonance threshold ($f_{res}$ > 1Hz and $Q$ > 1.05).

**c-d** Proportion of resonant cells overall (c) and stratified by subclass (d). Chi-square tests.

**e-g** $f_{res}$ (e), $f_{3\ dB}$ (f), and Q (g) compared across species ($n$ = 286 from 92 human donors; $n$ = 245 from 137 mice) and subclasses ($n_{LAMP5}$ = 253, $n_{VIP}$ = 108, $n_{PV}$ = 114, $n_{SST}$ = 56). (Linear mixed-effects (LME) models (Fixed: Species, Subclass, $V_m$; Random: Donor. Mann-Whitney U tests per subclass). Box plots: median and interquartile ranges. Open circles represent mouse recordings.

* $p < 0.05$, ** $p < 0.01$, *** $p < 0.001$

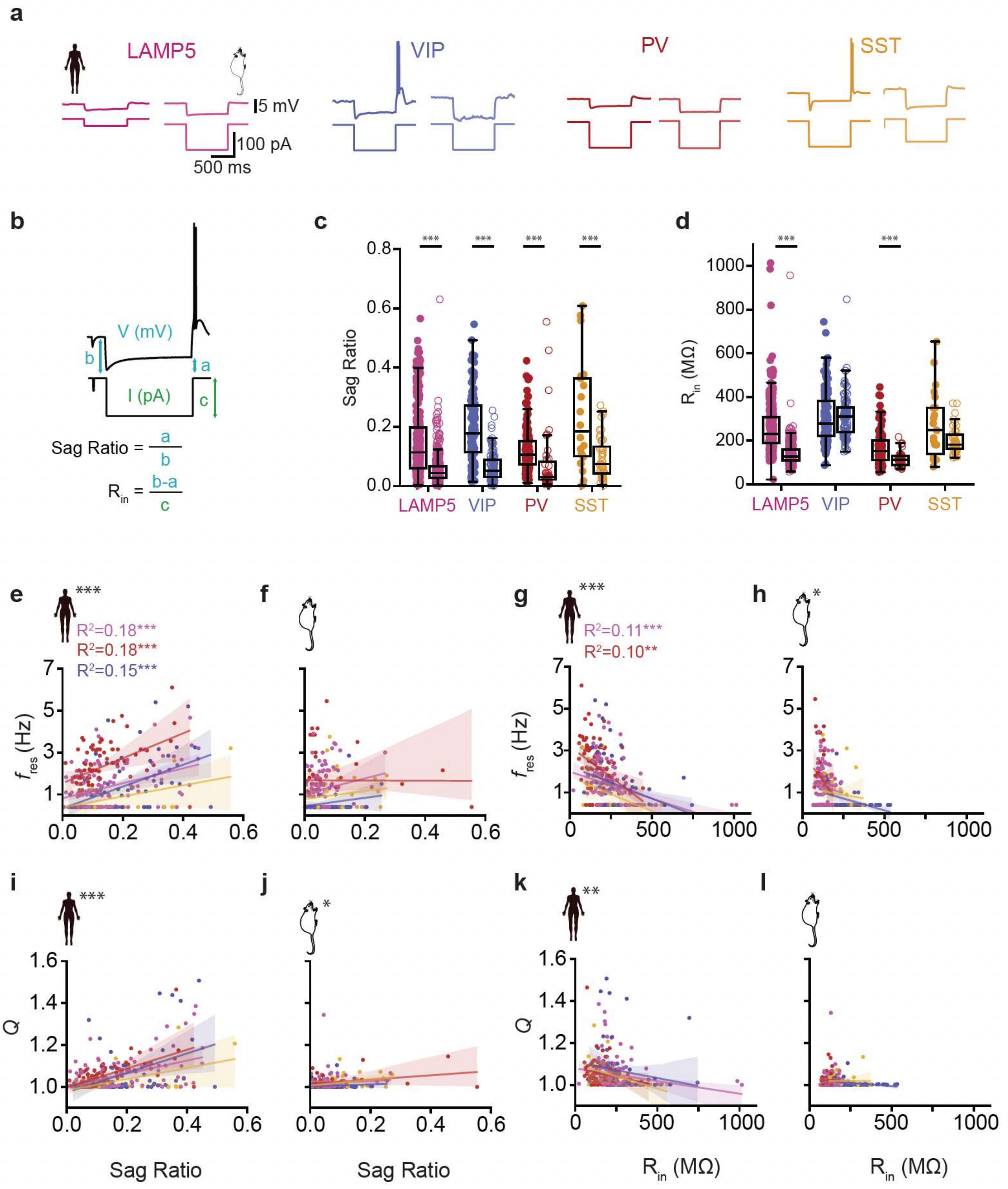

**Fig. 3 | Sag ratio and input resistance relate to strength and peak of resonance**

**a** Representative voltage responses to hyperpolarizing current injections.

**b** Schematic of sag ratio and input resistance calculations.

**c-d** Sag ratio (c) and input resistance (d) across species (Human, Mouse; $n$ = 541 from 130 human donors; $n$ = 619 from 289 mice) and subclasses ($n_{LAMP5}$ = 711, $n_{VIP}$ = 215, $n_{PV}$ = 169, $n_{SST}$ = 65).

(Linear mixed-effects (LME) models (Fixed: Species, Subclass, $V_m$; Random: Donor. Mann-Whitney U tests per subclass). Box plots: median and interquartile ranges.

**e-l** $f_{res}$ (e-h) and $Q$ (i-l) against sag ratio and input resistance per species (Human: $n$ = 287 from 92 donors, where $n_{LAMP5}$ = 135, $n_{VIP}$ = 59, $n_{PV}$ = 78, $n_{SST}$ = 15; Mouse: $n$ = *245 from 137 mice*, where $n_{LAMP5}$ = 118, $n_{VIP}$ = 49, $n_{PV}$ = 36, $n_{SST}$ = 42).

(Linear mixed-effects (LME) models (Fixed: Sag, Input resistance, Subclass; Random: Donor. Post-hoc analysis: LME per subclass).

* $p < 0.05$, ** $p < 0.01$, *** $p < 0.001$

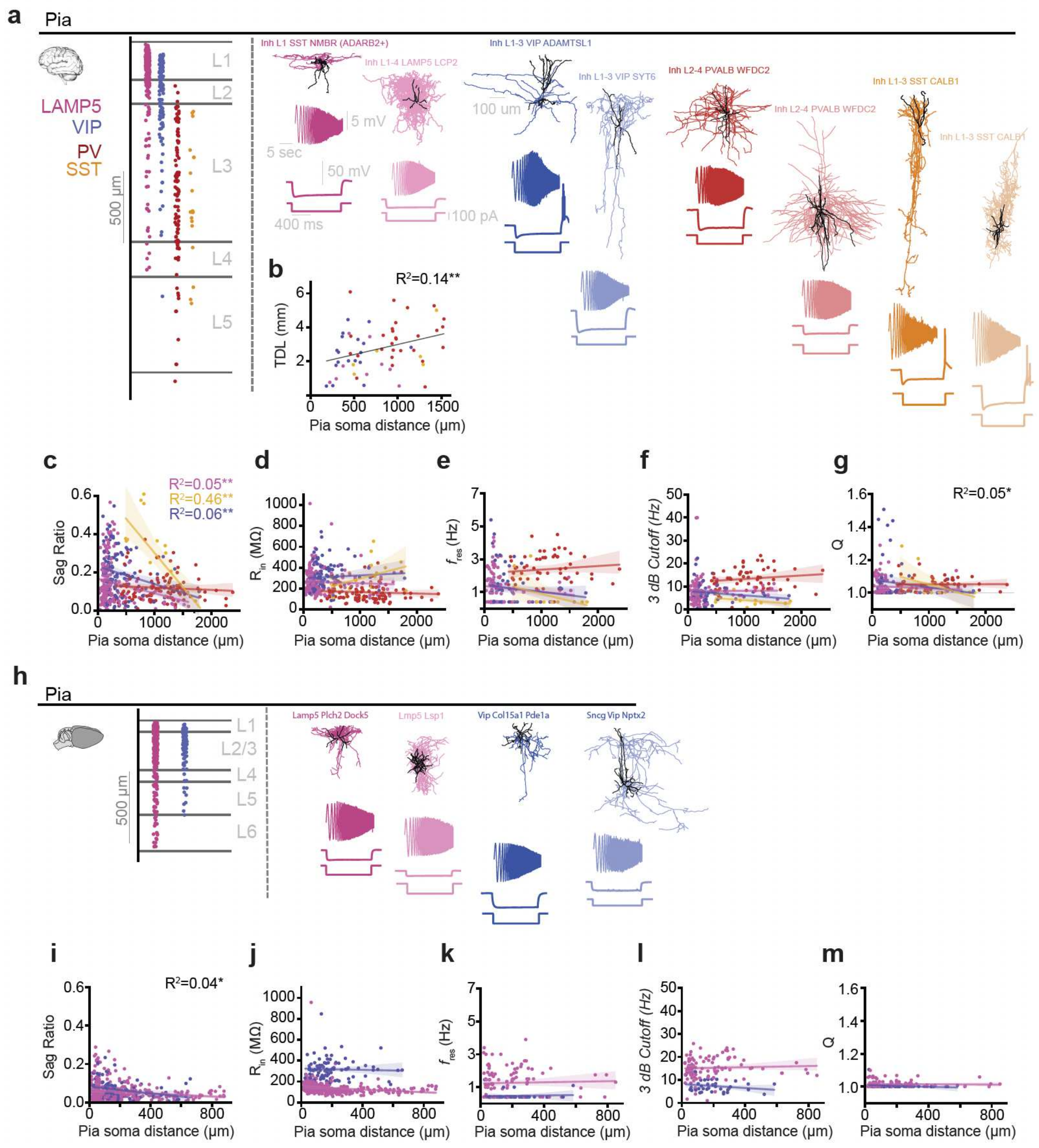

### Fig. 4 | Resonance features across cortical depth locations

**a** (Left) Interneuron cell body distances from pia plotted for LAMP5 (*n* = 241), VIP (*n* = 116), PV (*n* = 107), SST (*n* = 19) interneurons. (Right) Example morphologies of more (dark trace) and less (light trace) superficial cells per subclass in the human cortex. Examples taken from the MTG are plotted alongside corresponding chirp and subthreshold voltage traces per neuron.

**b** Pia-soma distance ($\mu$m) plotted against total dendritic length (TDL, *mm*). (Linear mixed-effects model. Fixed: pia-soma distance; Random: donor).

**c-g** Pia-soma distance plotted against passive ($n_{LAMP5}$ = 218, $n_{VIP}$ = 107, $n_{PV}$ = 106, $n_{SST}$ = 18; from 93 donors) and resonance-related membrane properties ($n_{LAMP5}$ = 110, $n_{VIP}$ = 56, $n_{PV}$ = 57, $n_{SST}$ = 11; from 64 donors) of interest in human acutely recorded interneurons. (Linear mixed-effects (LME) models (Fixed: pia-soma distance, subclass. Random: donor. Post-hoc analysis: LME per subclass).

**h** (Left) Interneuron cell body distances from pia plotted for LAMP5 *(n* = 396) and VIP *(n* = 91) interneurons. (Right) Example morphologies of more (dark trace) and less (light trace) superficial cells for mouse LAMP5 and VIP interneurons plotted alongside corresponding chirp and subthreshold voltage traces per neuron.

**i-m** Pia-soma distance plotted against passive ($n_{LAMP5}$ = 391, $n_{VIP}$ = 90; from 228 mice) resonance-related features ($n_{LAMP5}$ = 105; $n_{VIP}$ = 41; from 98 mice) of interest in mouse acutely recorded interneurons. (Linear mixed-effects (LME) models (Fixed: pia-soma distance, subclass. Random: donor).

*$p < 0.05$; **$p < 0.01$, ***$p < 0.001$

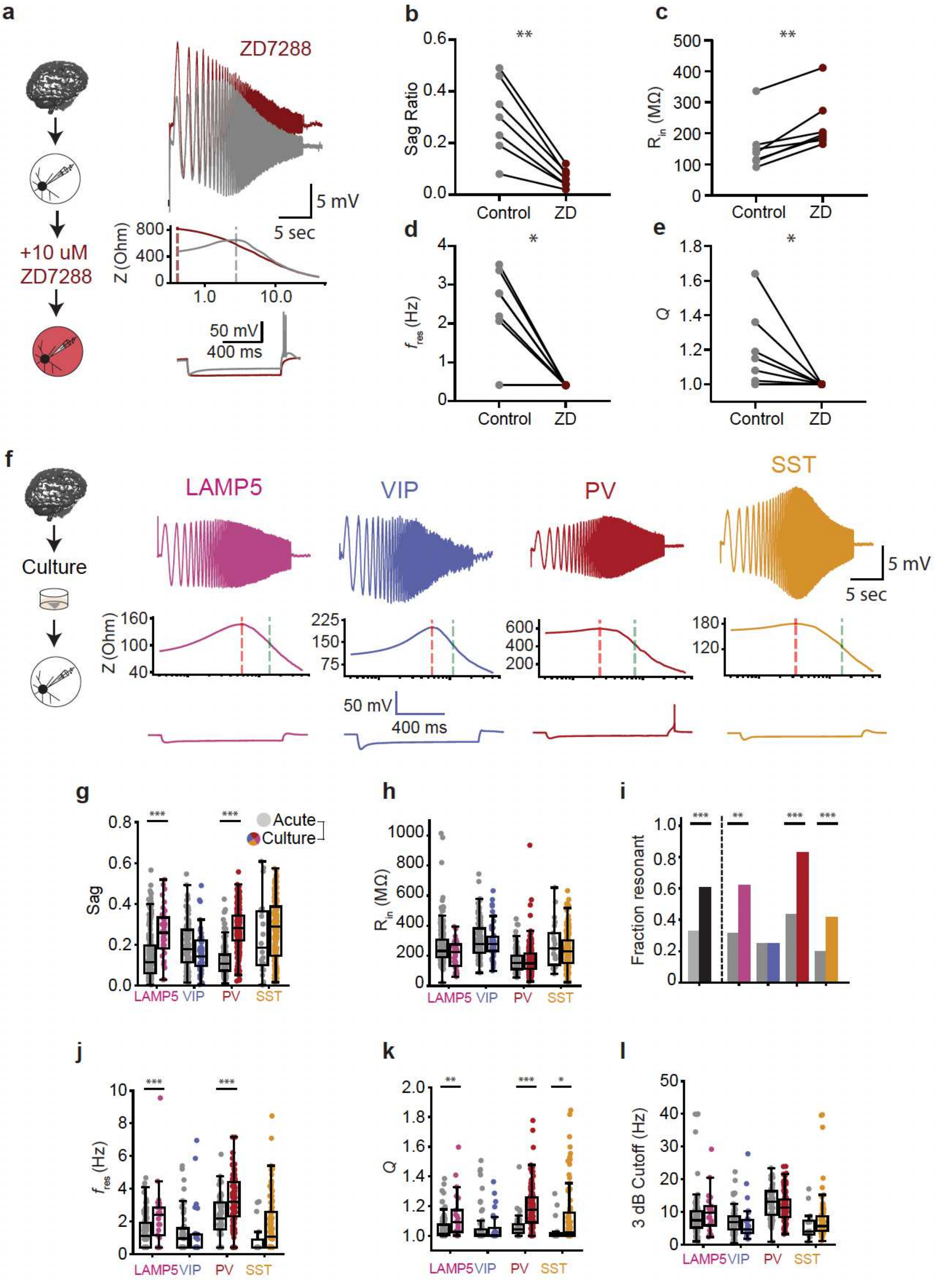

**Fig. 5 | Voltage sag properties support resonance in human interneurons**

a Bath application of 10 µM of ZD7288 in acutely recorded human cortical interneurons. Representative voltage trace in response to CHIRP stimulus before (grey) and after (red) ZD7288 application (*n* = 7, top), corresponding impedance profiles (middle) and voltage traces in response to hyperpolarizing current (-100 pA) injections (bottom). Dashed lines represent peak resonance frequencies.

b-e Sag ratio, input resistance, peak resonance frequency ($f_{res}$), and resonance strength ($Q$) values for each cell before and after ZD7288 application (Wilcoxon matched-pairs signed rank tests). Solid lines: repeated measures.

f Representative voltage responses to CHIRP stimulus in cultured LAMP5 (pink, *n = 21*), VIP (blue, *n = 28*), PV (red, *n* = 125), and SST (yellow, *n = 94*) interneurons with impedance profiles (middle) and voltage traces in response to hyperpolarizing current (-110 pA) injections (bottom). Red dashed lines: $f_{res}$. Green dashed lines: $f_{3\ dB}$.

g-h Sag ratio (g) and input resistance (h) values across subclasses ($n_{LAMP5}$ = 314, $n_{VIP}$ = 164, $n_{PV}$ = 405, $n_{SST}$ = 194) and preparations ($n_{Acute}$ = 541, $n_{Culture}$ = 536 from 192 donors).

(Linear mixed-effects (LME) models (Fixed: Preparation, Subclass, $V_m$; Random: Donor. Mann-Whitney U tests per subclass). Box plots: median and interquartile ranges.

i Proportions of resonant cells in acute and cultured interneurons per subclass. Chi-square tests.

j-l $f_{res}$ (j), $Q$ (k), and 3 dB cutoff frequencies (l) across subclasses ($n_{LAMP5}$ = 155, $n_{VIP}$ = 86, $n_{PV}$ = 201, $n_{SST}$ = 106) and preparations ($n_{Acute}$ = 286, $n_{Culture}$ = 262, from 145

donors). (Mann-Whitney U tests per subclass). Box plots: median and interquartile ranges.

* $p < 0.05$, ** $p < 0.01$, *** $p < 0.001$

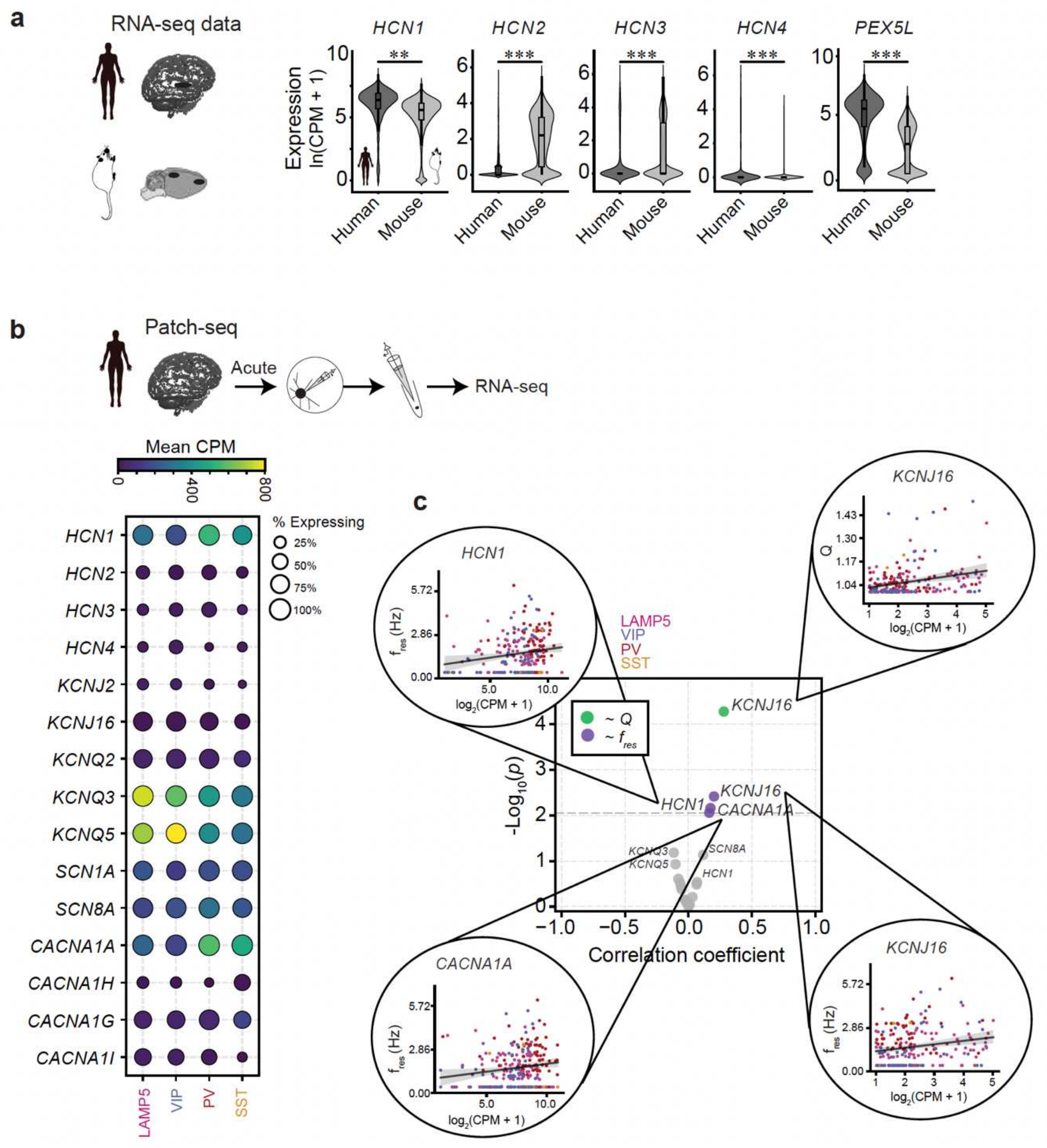

### Fig. 6 | Resonance-related gene enrichment in human cortical interneurons

**a** Violin plots showing counts per million (CPM) values of *HCN1-4* and *PEX5L* gene transcripts among interneurons in human middle temporal gyrus (MTG) (*n* = 4,164 cells; 8 donors), and mouse primary visual cortex (VISp) and anterior lateral motor cortex (ALM) (*n* = 10,534 cells, 210 animals).

(Linear mixed-effects (LME) models. Fixed: Species; Random: Donor. Significance annotations: BH-corrected *p*-values).

**b** Patch-seq expression levels (CPM) of resonance-related genes in human cortical interneurons across subclasses (LAMP5 *(n* = 256), VIP *(n* = 116), PV *(n* = 121), SST *(n* = 20)). Fill color and size represent mean CPM values and fraction of expressing cells per subclass, respectively.

**c** Gene expression (CPM > 1) across acutely recorded interneurons correlated against resonance parameters (*Q* and $f_{res}$). Pearson correlation coefficients are depicted against -$\log_{10}$ FDR-adjusted significance value per gene. Dashed line: significance threshold. Insets: Expression levels of significantly correlated genes (*KCNJ16*, *HCN1*, *CACNA1A*) plotted against resonance strength (*Q*) and resonance frequency ($f_{res}$).

*p < 0.05 **p < 0.01, ***p < 0.001

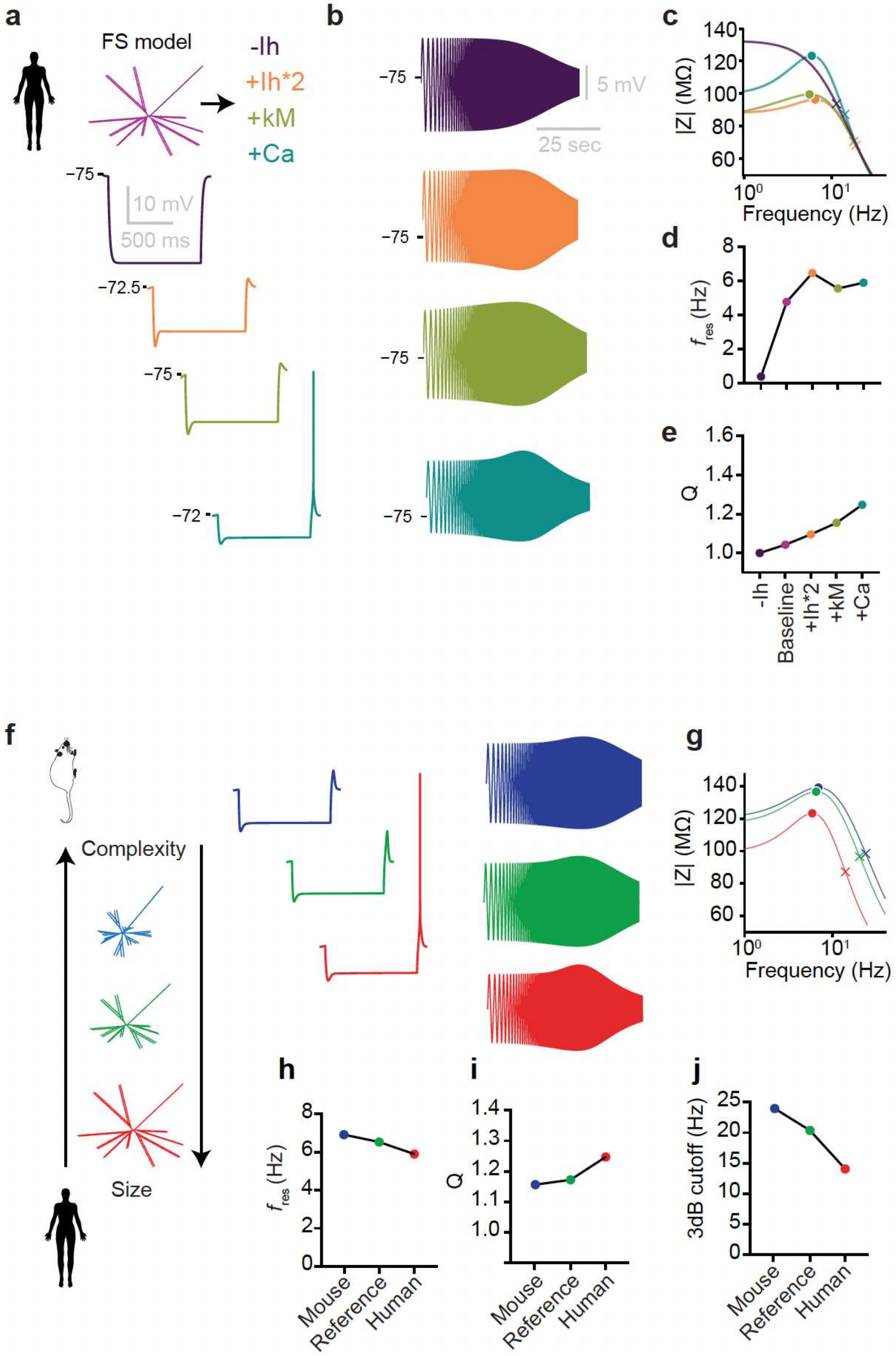

a
FS model
-Ih
+Ih*2
+kM
+Ca
−75
10 mV
500 ms
−72.5
−75
−72
b
−75
5 mV
25 sec
−75
−75
−75
c
|Z| (MΩ)
Frequency (Hz)
d
$f_{res}$ (Hz)
e
Q
-Ih
Baseline
+Ih*2
+kM
+Ca
f
Complexity
Size
g
|Z| (MΩ)
Frequency (Hz)
h
$f_{res}$ (Hz)
Mouse
Reference
Human
i
Q
Mouse
Reference
Human
j
3dB cutoff (Hz)
Mouse
Reference
Human

**Fig. 7 | Human morphological features are associated with sharper frequency tuning**

a-e Show results of active conductance manipulations in human-like FS interneuron model. Voltage traces in response to hyperpolarizing current injections (a) and logarithmic chirp stimulus (0.4 - 40 Hz; 65 s) (b) with corresponding impedance profiles (c). Peak resonance frequency ($f_{res}$) (d) and strength ($Q$) (e) values are depicted for each model.

f-j FS interneuron morphological features varied in dendritic size and complexity (left panel). Plotted are corresponding voltage responses to hyperpolarizing current injections (middle panel) and logarithmic chirp stimuli (0.4 - 40 Hz; 65 s) (right panel) and corresponding impedance profiles (g). $f_{res}$ (h), $Q$ (i), and 3 dB cutoff frequency (j) values are depicted for each model.

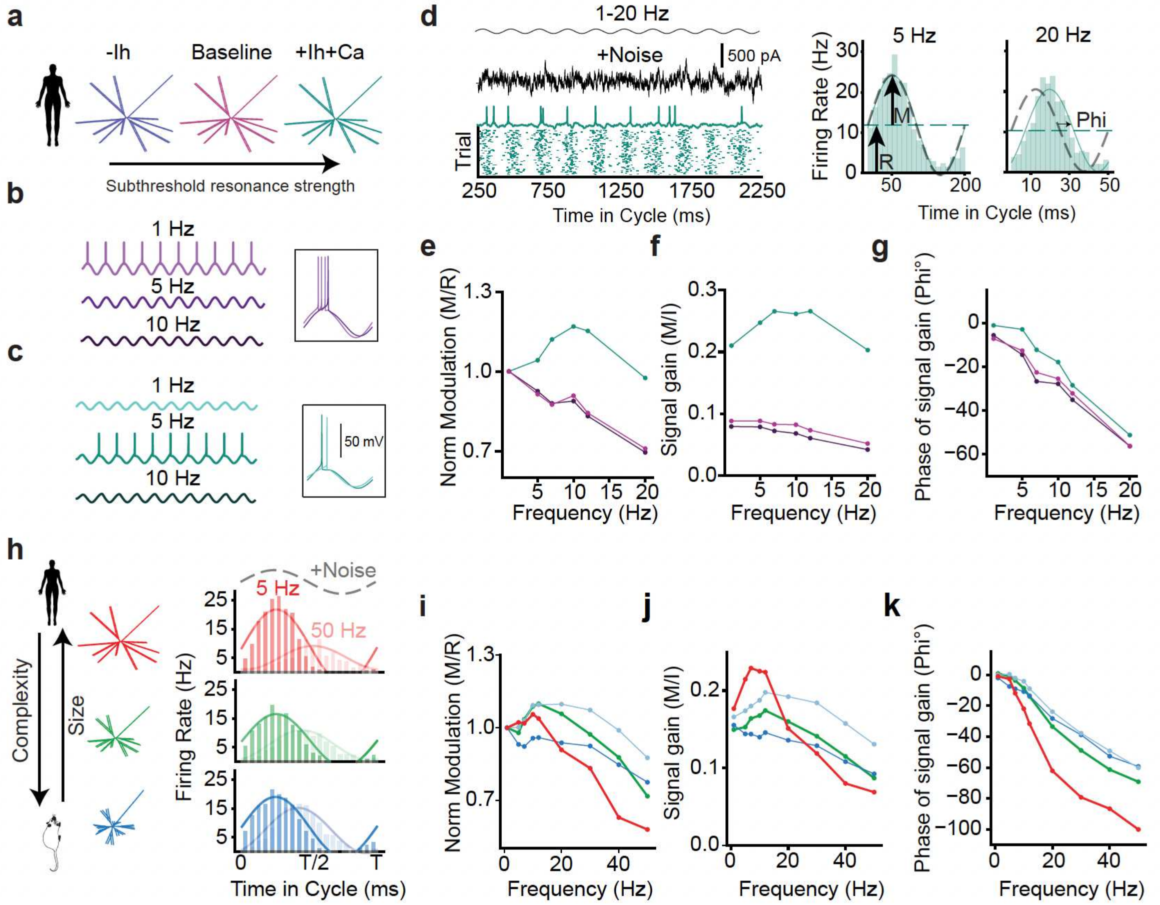

**Fig. 8 | From subthreshold to firing rate resonance**

**a** Experimental protocol.

**b-c** Voltage responses of a non-resonant (b) and a strongly resonant (c) interneuron to 1, 5, and 10 Hz sinusoidal stimuli (150 pA and 130 pA, respectively). Insets: 1 Hz and 5 Hz stimuli rheobase injections overlaid for comparison.

**d-g** Noisy signal detection protocol. Peristimulus histograms depict trial-averaged firing rates. Modulation index (M) and baseline excitability (R) and delay between peaks (Phi) are indicated (**d**). Resulting normalized modulation index (M/R) (**e**), signal gain (M/I) (**f**), and phase of signal gain (Phi) (**g**) values are given for each frequency stimulus for each model.

**h-k** FS-models with mouse-like and human-like idealized morphologies (**h**, left panel). Trial-averaged peristimulus histograms derived from voltage responses to 5 and 50 Hz sinusoidal current injections for comparison (**h**, right panel). Resulting normalized modulation index (M/R) (**i**), signal gain (M/I) (**j**), and phase of signal gain (Phi) (**k**) are given for each stimulus frequency for each model.

## Supplementary Files

This is a list of supplementary files associated with this preprint. Click to download.

- ShamugiaetalSupplementary2026.pdf
